# Function of *HIF-1α* in Regulating Sphingolipid Metabolism and Alleviating Oxidative Stress Damage in *Callosobruchus chinensis* Under Hypoxia

**DOI:** 10.3390/insects17090904

**Published:** 2026-08-28

**Authors:** Xiao Li, Sufen Cui, Zhichao Wan, Xuemei Sun, Yutong Liu, Xueqing Geng, Yujie Lu

**Affiliations:** 1 School of Grain Science and Technology, Jiangsu University of Science and Technology, Zhenjiang 212004, China; 231211803116@stu.just.edu.cn (X.L.); 18361816569@163.com (Z.W.); 18906498910@163.com (X.S.); luyjlyj71@just.edu.cn (Y.L.); 2 School of Biotechnology, Jiangsu University of Science and Technology, Zhenjiang 212004, China; liuyutong010203@163.com; 3 School of Agriculture and Biology, Shanghai Jiao Tong University, Shanghai 200240, China; xqgeng@sjtu.edu.cn

**Keywords:** hypoxia-inducible factor 1α, *Callosobruchus chinensis*, hypoxia, sphingolipid metabolism, oxidative damage

## Abstract

It has been suggested that hypoxia-inducible factor 1α (*HIF1α*) serves as a key regulatory factor in insect metabolism remodelling under hypoxia, in which lipid metabolism appears to play an important role. However, the underlying mechanism remains incompletely understood. In this paper, the function of *HIF1α* in regulating lipid metabolism under hypoxia in *Callosobruchus chinensis* (Coleoptera: Bruchidae), a typical stored-product insect, was investigated. Based on the amplification and cloning, the sequence of the *CcHIF1α* gene was analysed and found to be highly conserved throughout evolution. Then *HIF1α* expression in the adult *C. chinensis* was knocked down using RNA interference (RNAi) via feeding methods. After *HIF1α* knockdown, insects under hypoxic conditions exhibited higher mortality, along with decreased activities of four typical antioxidant enzymes (CAT, POD, GST, and SOD) and increased levels of two peroxide products (ROS and LPO), compared to those of the control. Moreover, comprehensive lipidomic analysis using UHPLC-QE–MS/MS revealed that lipid metabolism was reprogrammed, with significant changes in the levels of lipids such as SM, LPC, PI, SQDG, MAG, PE, ACar, and TAG. In particular, the increase in ceramide (Cer) or Cer-related lipids was observed to be associated with the production of peroxides. ELISA assays further verified the effect of *HIF1α* on changes in these characteristic lipids, such as the increase in PA and Cer and a decrease in TAG, PC and SM. Correspondingly, the expressions of the key genes (i.e., *SPTLC_1_*, *KDSR*, *SMPD_3_*, *SGPL_1_*, *DGAT*) involved in sphingolipid metabolism changed following *HIF1α* knockdown. These results are consistent with the possibility that *HIF1α*-mediated regulation of lipid metabolism may be important for insects facing hypoxic environments, and these findings could provide an in-depth understanding of insect hypoxia adaptation, thereby potentially contributing to the development of innovative pest management strategies.

## Simple Summary

*Callosobruchus chinensis* (Coleoptera: Bruchidae) is a typical stored-product insect that exhibits remarkable adaptability to hypoxic environments. Hypoxia-inducible factor 1α (*HIF1α*) is a key stress-responsive transcription factor in insects under low-oxygen conditions; however, its regulatory role in lipid metabolism remains poorly understood. This study, therefore, aimed to investigate the function of *HIF1α* in modulating lipid metabolism in *C. chinensis.* The full sequence of the *CcHIF1α* gene was cloned and characterized, revealing its highly conserved structure. After knockdown via interference (RNAi), insects showed higher mortality, decreased activities of typical antioxidant enzymes, and increased levels of reactive oxygen species (ROS) and lipid peroxidation products (LPO) under hypoxia. Moreover, lipid metabolism was reprogrammed, especially sphingolipid metabolism. Accordingly, the expression of the genes (i.e., *SPTLC_1_*, *KDSR*, *SMPD_3_*, *SGPL_1_*, and DGAT) involved was significantly altered. These results further prove the crucial role of *HIF1α* in mediating the lipid metabolism of insects under hypoxic stress conditions.

## 1. Introduction

Hermetic storage based on a low oxygen (<20% O_2_, hypoxia) and/or high carbon dioxide (hypercapnia) atmosphere in airtight containers is widely used for stored-product insect control [1,2]. Hypoxia impairs oxygen-dependent ATP synthesis, leading to the accumulation of reactive oxygen species (ROS) and subsequent oxidative damage in insects. This cascade disrupts essential physiological functions—including feeding, growth, and reproduction—and ultimately results in death. However, insects such as Coleoptera, Lepidoptera, Orthoptera, Diptera, and Isoptera have evolved remarkable hypoxia adaptability [2,3,4,5]. This is becoming a significant challenge during the sustainable application of hermetic storage technology.

It has been found that insects can survive hypoxic stress by employing various physiological and behavioural strategies, including metabolic remodelling, behavioural alterations, and adjustments to tissue structure, body size, lifespan, and fecundity [6,7,8,9,10]. Hypoxia-inducible factor 1 (HIF1), serves as a primary regulator hypoxic adaptation in insects. It functions as a heterodimeric DNA-binding complex composed of α and β subunits. Under normoxia, the *HIF1α* subunit is subject to O_2_-dependent hydroxylation, ubiquitination and proteasomal degradation. Under hypoxia, the oxygen availability is limited, and the protein is stabilized and forms the HIF1 transcription factor with *HIF1β* to activate the downstream genes for improving hypoxic adaptions [11,12,13,14]. Various reports have shown the important function of *HIF1α* as one of the important stress-responsive transcription factors in insect under hypoxia conditions [6,15]. In *Drosophila*, *HIF1α* (known as sima) can modulate growth and survival during periods of hypoxia [4]; HIF1α in mitochondria (Mito-HIF1α) can attenuate apoptosis induced by exposure to hypoxia or H_2_O_2_-induced oxidative stress, reducing the production of ROS [16]. However, the detailed mechanisms are not fully understood.

Our previous studies demonstrated that dozens of genes and metabolites associated with lipid metabolism, especially sphingolipid metabolism, were significantly altered in *C. chinensis* under hypoxic compared to normoxic conditions [17]. Sphingolipids are not only essential structural components of cells but also act as key signalling molecules [18,19]. Of them, ceramides, composed of sphingosine and fatty acid, function as the important messengers involved in cell growth inhibition and apoptosis, and they interact with several pathways to promote insulin resistance, oxidative stress, and inflammation [20]. Hypoxia activates the neutral sphingomyelinases (nSMases), which are key enzymes in ceramide synthesis, enhancing the production of ceramide [21]. Ceramide accumulation promotes mitochondrial ROS production and exacerbates oxidative stress. whereas ROS can activate sphingomyelinases, thereby further stimulating ceramide accumulation [22,23,24].

*HIF1α* has been reported to participate in ceramide generation by directly regulating *SMPD_3_*, the gene encoding neutral sphingomyelinase. The injection of lentivirus *SMPD_3_* in epididymal adipose tissue reversed the decrease in ceramides in adipocytes and eliminated the improvements in atherosclerosis in the adipocyte HIF1α-deficient mice [20]. Ceramide synthesis involves multiple interconnected pathways, including the de novo pathway, the sphingomyelinase pathway—in which sphingomyelin is hydrolysed by sphingomyelinases—and the salvage pathway, where ceramide synthase reutilizes long-chain sphingoid bases to generate ceramide [22]. Therefore, the role of *HIF1α* in ceramide generation is still unclear.

In this study, the function of *HIF1α* in the regulation of sphingolipid metabolism in *Callosobruchus chinensis* (Linnaeus) (Coleoptera: Bruchidae) (*CcHIF1α*), a typical stored-product insect, under hypoxia was further investigated. The full-length sequence of *CcHIF1α* was cloned and analysed. siRNA-loaded chitosan nanoparticles were synthesized for *HIF1α* interference. After *CcHIF1α* knockdown using RNA interference (RNAi), the changes in adult *C. chinensis* under hypoxia, including mortality, oxidative stress response, lipid metabolism, and the expression of related genes, were assessed. These findings will further advance our understanding of the regulatory mechanisms of *HIF1α* in insect hypoxic adaptation. They also provide potential molecular targets for developing novel pest management strategies, which may help improve the efficacy of hermetic storage for controlling stored-product insects in the future.

## 2. Materials and Methods

### 2.1. Insect Rearing and Hypoxia Treatment

The *C. chinensis* (L.) used in this study were obtained from the laboratory of the College of Plant Protection, Shanxi Agricultural University. They were maintained on mung bean, *Vigna radiata* (L.), for over five generations. *C. chinensis* were fed with mung bean, *Vigna radiata* (L.), at density of 2–3 insect per seed and maintained in an environmental controlled chamber at 28 °C and 65% RH. The *C. chinensis* adults were used in this study. Briefly, adult insects (newly emerged, about 1~3 days) were transferred into new feeding bottles containing pre-mixed gases (hypoxia, 2% O_2_ + 98% N_2_) according to the methods used in our previous reports [17]. Gas concentrations were measured using a Checkpoint analyser (Dansensor A/S, Ringsted, Denmark). The inside of the feeding bottle neck was coated with Daflon to preventing insects crawling out. Each experiment was performed with three replicates. After hypoxia treatment, the samples from each group were collected, frozen immediately in liquid nitrogen, and stored at −80 °C for subsequent experiments. The duration of hypoxia exposure was varied according to the subsequent experimental design.

### 2.2. Total RNA Extraction and Real-Time Quantitative (RT-qPCR)

Total RNA was extracted from 2–4 adult (approximately 5 mg) insects, treated with 24 h of hypoxia, using Column Total RNA extraction reagent (Sangon, Shanghai, China). Residual genomic DNA was removed using an RNA kit GD3011 (BioTek, Beijing, China) and dissolved in RNase-free water. The integrity of the RNA was checked using an Agilent 2100 Bioanalyzer system with an RNA 6000 Nano kit (Agilent Technologies, Palo Alto, CA, USA). Purified RNA samples were stored at −80 °C.

A total of 1 μg of total RNA was reverse-transcribed to cDNA using the PrimeScript™ RT reagent Kit with gDNA Eraser (Takara Bio, Kusatsu, Japan). The relative transcription levels of *HIF1α* and its downstream genes were amplified using SYBR Premix Ex Taq™ II Kit (Takara Bio, Kusatsu, Japan) in a CFX connect Real-Time system (Bio-Rad, Hercules, CA, USA). β-actin was chosen as a reference to normalize the mRNA levels between the treatment and control groups. Primer specificity was examined by dissociation curve analysis; fold-change values were calculated using the 2^−ΔΔCt^ method [25,26]. Each experiment was performed in triplicate with three technical replicates. The primers for *CcHIF1α* and downstream genes selected for RT-qPCR are presented in Table S1.

### 2.3. CcHIF1α Cloning and Vector Construction

The conserved sequence of *C. chinensis HIF1α* (*CcHIF1α*) was determined via RNA sequencing. Based on the conserved sequence of *CcHIF1α*, a pair of joint primers (F1 and F2) and a pair of specific primers (R1 and R2) were designed for full length cDNAs for the *HIF1α* subunit (Table S2). Nested PCR was performed with these two pairs of degenerate primers.

5′ RACE and 3′ RACE cDNA template synthesis was performed using a Revert Aid First Strand cDNA Synthesis Kit (Thermo Fisher, Waltham, MA, USA). The 5′ RACE cDNA template was used for terminal deoxynucleotidyl transferase tailing reactions, which were performed using a kit, according to the manufacturer’s protocol. The initial PCR amplification of terminal deoxynucleotidyl transferase-tailed 5′ RACE cDNA was performed with specific primers (*HIF1α*5′-R1 and *HIF1α*5′-F1) using the TAQ Plus DNA polymerase PCR system (Tiangen, Beijing, China), and the 3′ RACE cDNA template was amplified. The primary PCR amplification of terminal deoxynucleotidyl transferase-tailed 3′ RACE cDNA was performed with the primers *HIF1α*3′-R1 and *HIF1α*3′-F1 using a TAQ plus DNA polymerase PCR system (Tiangen, Beijing, China). The RACE segments were purified using a Universal DNA Recovery Kit (Tiangen, Beijing, China), and then subcloned into a vector and sequenced.

Total RNA was extracted from adult *C. chinensis*, and the full-length *HIF1α* cDNA was obtained by reverse transcription using a PrimeScript™ RT Reagent Kit (Takara Bio, Kusatsu, Japan). The 5′- and 3′-end sequences, together with the conserved *HIF1α* sequence, were assembled to obtain the complete full-length *HIF1α* cDNA sequence. Gene-specific primers (Table S2) were then used to amplify the full-length *HIF1α* ORF by PCR. The PCR products were resolved on a 1% agarose gel. Positive clones were identified using a seamless assembly cloning kit (CloneSmarter, Houston, TX, USA). The amplified *HIF1α* fragments were subsequently subcloned into the pET-30 vector, and the recombinant plasmids were verified by restriction enzyme digestion and nucleotide sequencing.

### 2.4. Sequence Analysis and Phylogenetic Tree Construction

The open reading frame (ORF) of *CcHIF1α* was identified using DNAMAN 9 software (Lynnon Biosoft, Vaudreuil-Dorion, QC, Canada). Protein similarity and homology analyses were performed using NCBI BLAST 2.16.0 https://blast.ncbi.nlm.nih.gov/Blast.cgi (accessed on 15 April 2024). Multiple sequence alignments of protein sequences were generated using ClustalW 2.1 http://www.genome.jp/tools/clustalw/ (accessed on 16 April 2024), and the resulting alignments with high similarity were visualized using BoxShade software 3.3.1. A phylogenetic tree was constructed using MEGA 11 using the neighbour-joining method and 1000 bootstrap replicates. The GenBank accession numbers of the *HIF1α* protein sequences used for sequence alignment and phylogenetic analysis are listed in Table S3 and Table S4, respectively.

### 2.5. siRNA and Chitosan-siRNA (CS-siRNA) Nanoparticles Synthesis

For RNA interference experiments, siRNA targeting *CcHIF1α* was synthesized, and siRNA targeting *GFP* was also synthesized as a negative control. Both siHIF1α and siGFP were purchased from GenePharma (Suzhou, China). The siRNA target sequences are listed in Table S5. The purity and integrity of the siRNA were examined using agarose gel electrophoresis and a NanoDrop™ Eight spectrophotometer (Thermo Fisher Scientific, Waltham, MA, USA).

Chitosan/siRNA nanoparticles were prepared according to previous methods, with slight modifications [27,28]. Briefly, 2 mg of chitosan (CS) was dissolved in 1 mL of acetic acid, and the pH of the solution was adjusted to 5.5. Subsequently, 200 μg of siRNA and 800 μg of sodium tripolyphosphate (STPP) were dissolved in the CS solution with sonication assistance for 3 min, followed by heating at 55 °C for 1 min and vortexing for 1 min. The mixture was incubated at room temperature for 1 h to allow the RNA molecules to be adsorbed into the CS-STPP microcapsules during particle synthesis. After centrifugation at 13,000 rpm for 10 min and two washes with deionized water, the microcapsules were collected. The encapsulation efficiency of the RNA was determined using UV–Vis spectroscopy at 260 nm. After air-drying, the CS-*siHIF1α* and CS-*siGFP* nanoparticles were obtained and stored at −80 °C for subsequent experiments.

### 2.6. RNA Interference Assays

CS-*siHIF1α* was resuspended with 2 mL nuclease-free water. Then mung beans used were pre-soaked with CS-*siHIF1α* solution, then dried under normoxic conditions for 24 h. After 24 h starvation, one-day-old *C. chinensis* adults were selected and fed on the mung beans containing CS-*siHIF1α* (4–5 adults per seed). 24 h later, adult insect were transferred to the hypoxic environment. Insects treated with CS-*siGFP* were set as the control. All experiments were replicated three times. For each biological replicate, a total of 300 adults (150 in the CS-*siGFP* control group and 150 in the CS-*siHIF1α*group) were used, amounting to 900 adults in total across all three replicates. The efficiency of RNAi was determined using RT-qPCR and Western blotting at 48 h of hypoxia exposure. Survival probability was assessed daily until all insects died. The expression of *HIF1α* was determined daily using RT-qPCR during the 7-day hypoxia exposure period.

### 2.7. Western Blots Analysis

Approximately 20–25 adult (weighing about 50 mg) insects were mixed with 505 μL of Tissue Total Protein Lysis Buffer (Sangon, Shanghai, China) in 1.5 mL screwcap microtubes. The samples were crushed using a TissueLyser (Qiagen, Hilden, Germany). After being vortexed for 1 min, the samples were stored at 4 °C for 30 min to extract metabolites, followed by centrifugation at 12,000× *g* for 10 min, and the protein was obtained. The protein concentration was detected using a BCA kit (Jiancheng, Nanjing, China). A total of 40 μg of protein per sample were separated on 12% sodium dodecyl sulphate polyacrylamide gel and subsequently transferred to PVDF membranes, which were sealed with skim milk powder for 2 h and incubated overnight at 4 °C with anti-*HIF1α* rabbit polyclonal antibody D262108-0025 (Sangon, Shanghai, China) and α-Tubulin rabbit Polyclonal antibody AF5012 (Beyotime, Shanghai, China). The membranes were washed five times and then incubated with horseradish enzyme-labelled goat anti-rabbit IgG secondary antibody at room temperature for 2 h. The membranes were washed with TBST (Sangon, Shanghai, China) 5 times. ECL enhancer (Servicebio, Wuhan, China) and stable peroxidase solution (1:1) were mixed and applied to the membrane. After a few minutes, when fluorescent bands appeared, excess substrate was removed with filter paper, the membrane was covered with plastic wrap, and it was expose to X-ray film. The film was developed, fixed, rinsed, and quantified with Image-Pro Plus v7.1, using α-tubulin as the loading control [29].

### 2.8. Oxidative Stress Response Analysis

The reactive oxygen species (ROS) levels, lipid peroxidation (LPO) content, and the activities of antioxidant enzymes (CAT, SOD, POD, and GST) in C. chinensis adults were measured using commercial reagent kits (Jiancheng, Nanjing, China) in accordance with the manufacturer’s instructions. Briefly, 30 adult *C. chinensis* were fed with CS-*siHIF1α* for 24 h, then transferred to hypoxic conditions, which were maintained for 48 h. Next, 50 mg of sample were collected, homogenized in 0.9% NaCl solution (1:9, *w*/*v*), and centrifuged at 2500 rpm and 4 °C for 10 min. The resulting supernatant was collected for oxidative stress response assays. Controls were treated with CS-*siGFP*. All experiments were performed in triplicate.

### 2.9. Lipidomic Analysis by UHPLC-QE–MS/MS

Lipids were extracted according to the previous method, with slight modifications [30]. Briefly, 50 adult *C. chinensis* were fed with CS-*siHIF1α* for 24 h, then transferred to hypoxic conditions and held for 48 h. Then, the insects were collected and frozen at −80 °C for the lipidomic analysis. A total of 25 mg of samples were placed in a 2.0 mL EP tube containing 800 μL of pre-cooled mixed solvent (H_2_O, methyl tert-butyl ether, and methanol at a ratio of 2:5:1 *v*/*v*/*v*). The samples were vortexed for 30 s and homogenized using a mechanical grinder (JXFSTPRP-24, Shanghai Jingxin, Shanghai, China) at 35 Hz for 4 min; then, ultrasound treatment was performed in a water bath at 0 °C for 5 min, repeated three times. The samples were then transferred to a −40 °C refrigerator for 1 h, followed by centrifugation at 3000 rpm, 4 °C for 15 min. The supernatant was collected, and 300 μL was transferred to a new EP tube for vacuum freeze-drying. The dried samples were resuspended in 200 μL of dichloromethane-methanol solvent (1:1, *v*/*v*) by sonication in an ice water bath for 10 min, followed by centrifugation at 12,000 rpm for 15 min at 4 °C. A total of 100 μL of supernatant was used for LC–MS analysis. A quality control (QC) sample was prepared with 20 μL of each sample’s supernatant, which were simultaneously analysed for confirming the accuracy of the experimental data. Controls were treated with CS-*siGFP*. The experiments were performed in six replicates.

LC–MS analyses were performed using a UHPLC system (Vanquish, Thermo Fisher Scientific, Waltham, MA, USA) equipped with a Kinetex C18 column (2.1 × 100 mm, 1.7 μm, Phenomenex, Torrance, CA, USA). Mobile phase A comprised H_2_O/acetonitrile (4:6, *v*/*v*) with 10 mM ammonium formate. Mobile phase B comprised isopropanol/acetonitrile (9:1, *v*/*v*), which was added into 50 mL of 10 mM ammonium formate for every 1000 mL of mixed solution. The analysis was carried out with gradient as follows: 0–1.0 min, 40% B; 6.3–8.6 min, 85% B; 8.7–9.3 min, 100% B; 9.4–12.0 min, 40% B. The injection volume was 2 μL. The column and auto-sampler temperatures were set to 55 °C and 4 °C, respectively, and the injection volume was 2 μL (for both positive- and negative-ion modes).

To acquire MS/MS spectra, an Orbitrap Exploris 120 mass spectrometer (Orbitrap MS, Thermo Fisher Scientific, Waltham, MA, USA) was used in data-dependent acquisition (DDA) mode, managed using acquisition software (Xcalibur 4.7, Waltham, MA, USA, Thermo). In this mode, the software continuously evaluated the full-scan MS spectrum. The electrospray ionization source conditions were set as follows: sheath gas flow rate of 30 Arb, auxiliary gas flow rate of 10 Arb, capillary temperature of 320 °C (positive-ion mode) or 300 °C (negative-ion mode), full MS resolution of 60,000, MS/MS resolution of 15,000, collision energy of 15/30/45 in the NCE mode, and spray voltage of 3.8 kV (positive-ion mode) or −3.4 kV (negative-ion mode).

Using ProteoWizard software 3.x, the raw data files were converted into mzXML format through the “msconvert” program. The CentWave algorithm in XCMS 4.10.1 was used for peak detection, peak extraction, peak comparison, and peak integration with the following parameters: minfrac to 0.5 and cutoff to 0.3. Lipids were identified using the LipidBlast library, which was developed using R software R 4.4.0 and based on XCMS. Differentially abundant lipids between the CS-*siHIF1α* and CS-*siGFP* groups were selected based on the following criteria: a *p*-value < 0.05 from a Student’s *t*-test and a Variable Importance in the Projection (VIP) score > 1.0, derived from the OPLS-DA model. Only lipids meeting both criteria were considered as significantly altered and subjected to further analysis.

### 2.10. KEGG Annotation and Enrichment Analysis

The metabolites were annotated by the HMDB 4.0 database (http://www.hmdb.ca/) and the Kyoto Encyclopaedia of Genes and Genomes (KEGG) database (https://www.kegg.jp/). Then, the identified metabolites were annotated by referencing the KEGG compound database and linked to KEGG pathway database http://www.kegg.jp/kegg/pathway.html (accessed on 20 April 2024) to display the roles in different biological processes. To ensure a more comprehensive and biologically relevant interpretation of the lipidomic data, a manual curation of the KEGG pathways was conducted. The pathways were further analysed through metabolites set enrichment analysis, and the relative importance was established using a hypergeometric test at *p*-value < 0.05.

### 2.11. Characteristic Lipid Content Analysis

The contents of characteristic lipids including ceramide (Cer), palmitic acid (PA) content, sphingomyelin (SM), phosphatidylcholine (PC), and triglyceride (TAG) were assessed using reagent kits (Sango, Shanghai, China; Lianzu, Shanghai, China), according to manufacturer’s instructions. Briefly, 30 adult *C. chinensis* were fed on mung beans with CS-*siGFP*, CS-*siHIF1α*, FG-4592 (HIF activator), or PX-478 (*HIF1α* inhibitor) for 24 h, then transferred to hypoxic conditions; 48 h later, the sample was collected, immediately frozen in liquid nitrogen and stored at 80 °C for subsequent experiments. A total of 20–25 adult insects (approximately 50 mg) were homogenized in 0.9% NaCl solution (1:9, *w*/*v*) and centrifuged at 2500 rpm and 4 °C for 10 min. The resulting supernatant was used for the lipid content examination. All experiments were performed in triplicate.

### 2.12. Data Analysis

Statistical significance (Tukey’s test, independent *t*-test and one-way ANOVA, *p* < 0.05) was determined using IBM SPSS Statistics 26. The VIP value, PCA and OPLS-DA were analysed using SIMCA 14.0 software [31]. Survival data were analysed using the Kaplan–Meier method, and differences between groups were evaluated by the log-rank (Mantel–Cox) test. Statistical analyses were performed using GraphPad Prism 10 and Origin 2021 software. Values are presented as mean ± SM. The threshold of significant was <0.05 (*p* < 0.05).

## 3. Results

### 3.1. The C. chinensis HIF1α Subunit (CcHIF1α) Exhibits High Sequence and Domain Structure Similarity with Homologues

**Figure 1:**
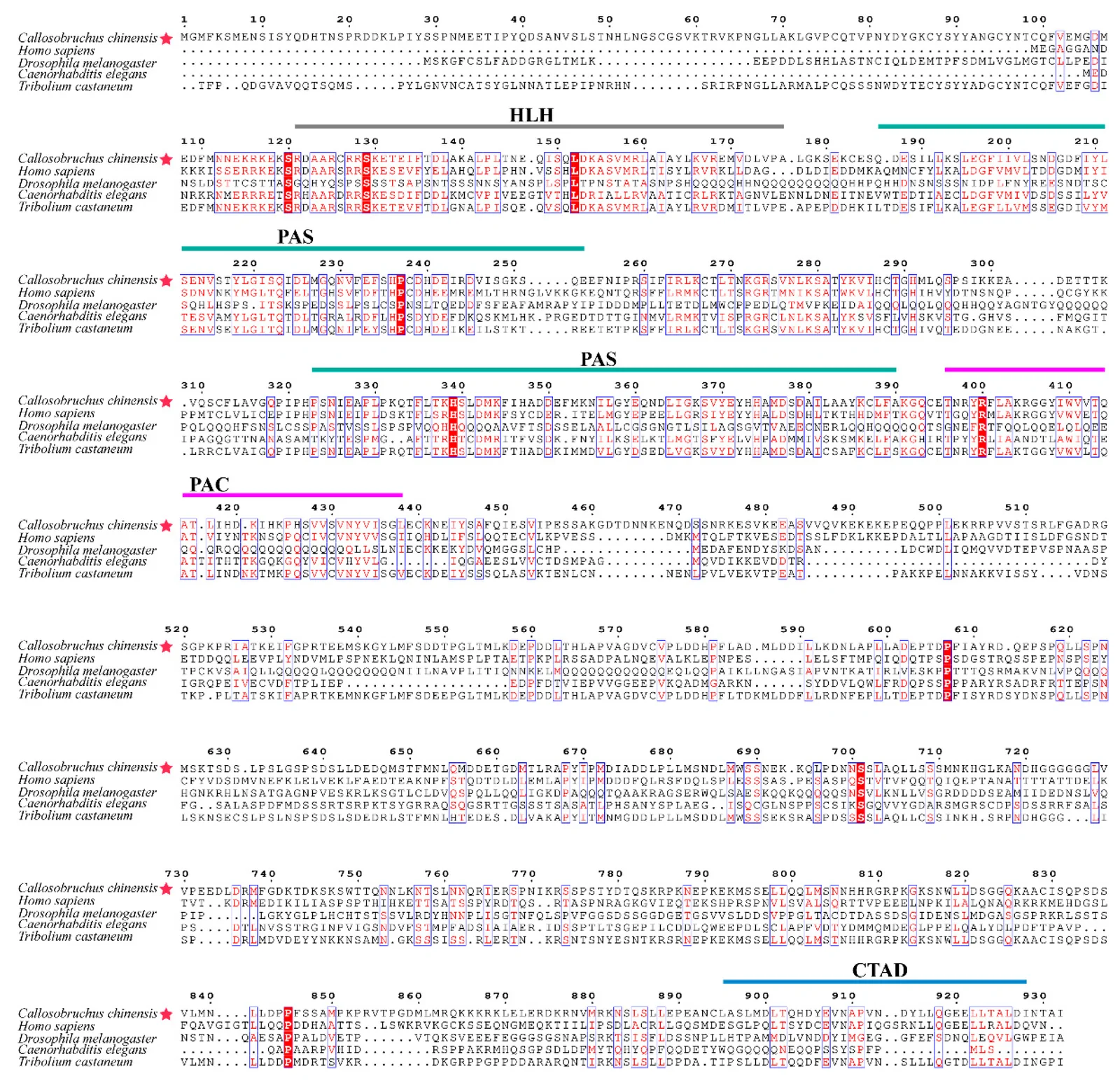
Sequence alignment of *HIF1α*. Full-length amino acid sequences from *C. chinensis*, *H. sapiens*, *Drosophila*, *C. elegans* and *T. castaneum* are aligned by ClustalX 2.1 and edited by ESPript 3.0. The Helix–loop–helix (HLH) domain, Per-Arnt-Sim (PAS) domain, PAS-associated C-terminal (PAC) and C-terminal transactivation domain (CTAD) regions are marked with lines above the sequence. Strictly identical residues are highlighted in white letters with a red background. Residues with similar physicochemical properties are shown in red letters. Alignment positions are framed in blue if the corresponding residues are identical or similar. The accession numbers of HIF1α protein sequence used and their identity are presented in Table S3.

To investigate the role of *CcHIF1α* in regulating *C. chinensis* response to hypoxia, the putative subunits sequence was cloned using RAC techniques (Table S6). These sequence data have been submitted to the GenBank databases under accession number PX427685. The full-length cDNA of *CcHIF1α* (3144 bp) contains an open reading frame (ORF) that encodes 931 amino acids. It contains a basic helix–loop–helix domain (bHLH), two Per-Arnt-Sim domains (PAS), a Per-Arnt-Sim-associated C-terminal domain (PAC), and a C-terminal transactivation subunit (CTAD). The sequence was aligned with the *HIF1α* from *Drosophila*, *C. elegans*, *human* and *T. castaneum*, which showed 52% identity with the templates of *HIF1α* from *Drosophila*, 30% from *C. elegans*, 49% from *H. sapiens* and 61% from *T. castaneum.* The amino acid sequence alignment of protein is shown in Figure 1, Table S3. These subunits share high sequence similarity and domain structure with homologues from *H. sapiens*, *Drosophila*, *C. elegans* and *T. castaneum*.

**Figure 2:**
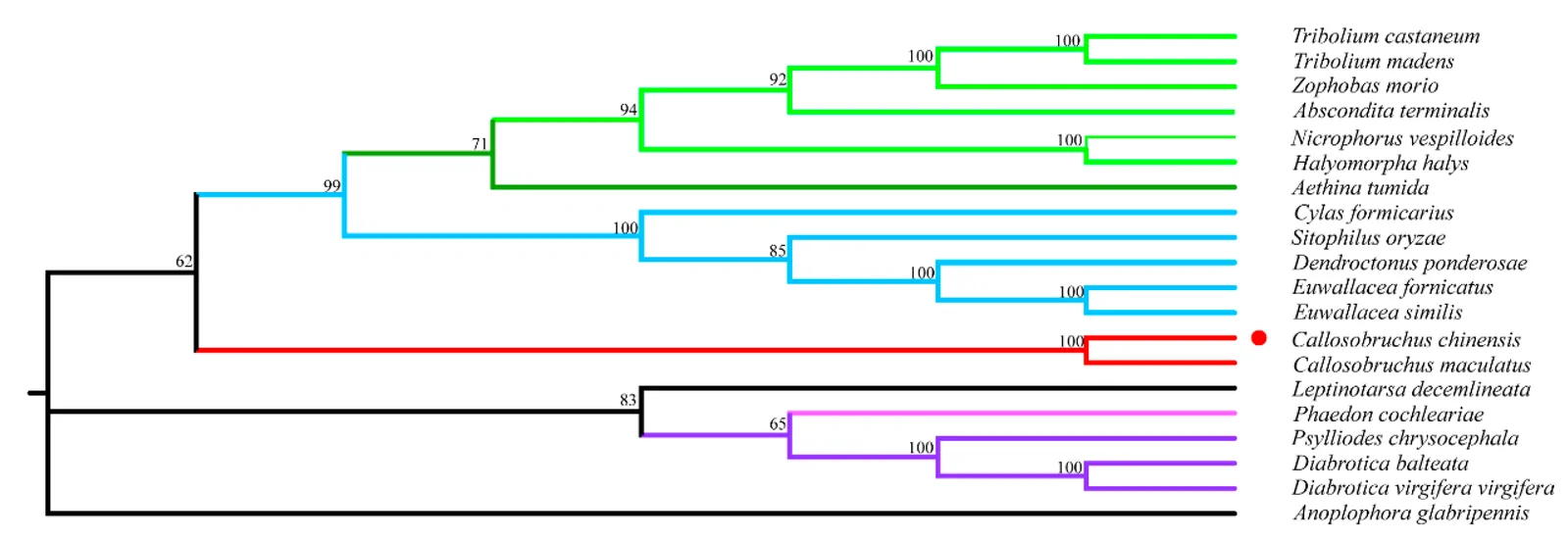
Phylogenetic analysis of *HIF1α* amino acid sequences from 20 coleopteran insects. HIF1α amino acid sequences from *C. chinensis* (*CcHIF1α*) and 19 other coleopterans. The unrooted consensus tree was constructed using the MEGA 11.0 program with neighbour-joining phylogeny and the p-distances model. The bootstrap values are calculated by 1000 replicates and listed at each node. Bootstrap values > 50% are shown on the branches. The accession numbers of the HIF1α protein sequence used, along with the reliability, are presented in Table S4.

The neighbour-joining phylogenetic tree was constructed with *HIF1α* from *C. chinensis* and 19 of other coleopterans, such as *C. maculatus* (Coleoptera: Bruchuidae), *Anoplophora glabripennis* (Coleoptera: Cerambycidae), *T. castaneum*, and *T. madens* (Coleoptera: Tenebrionidae). There was strong bootstrap support for the division of Coleoptera *HIF1α* homologs into one major phylogenetic branch with two distinct clusters, each containing several *HIF1α* homologs from different species. The *CcHIF1α* showed about 61.95% average sequence identities matching those of other Coleopteran *HIF1α*. The phylogenetic tree analysis showed that *CcHIF1α* is clustered most closely with their orthologous *HIF1α* of *C. maculatus* (Figure 2, Table S4).

### 3.2. HIF1α Improved the Survival Ability of C. chinensis Adults Under Hypoxia

**Figure 3:**
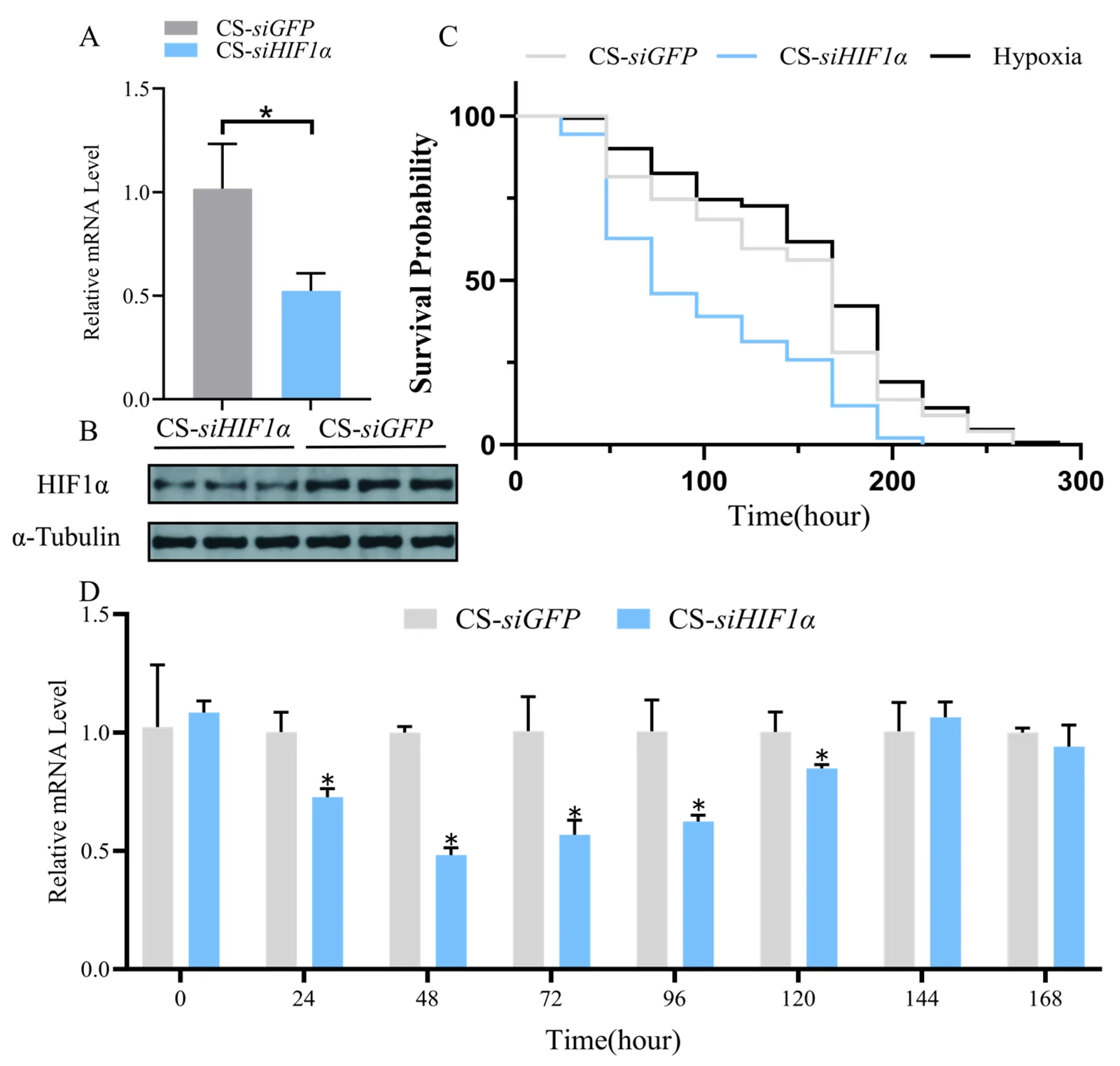
Effect of *HIF1α* on survival of *C. chinensis* adults under hypoxia. Expression of *CcHIF1α* at mRNA (**A**) and protein (**B**) levels following 48 h of hypoxia exposure. (**C**) Kaplan–Meier survival curves. (**D**) Relative mRNA level of *CcHIF1α* following 168 h of hypoxia exposure. The significant differences are denoted with * *p* < 0.05 using an independent *t*-test. The CS-*siHIF1α* group (*n* = 100 per replicate, three independent replicates) showed significantly reduced survival compared with that of the CS-*siGFP* control group (log-rank test, *p* < 0.0001). The bars represent mean ± SE of three repeats. Hypoxia: 2% O_2_ + 98% N_2_.

For the RNA interference experiment, CS-*siHIF1α* and CS-*siGFP* nanoparticles were prepared, with the loading efficiency of 53.4% and 51.9%, respectively (Figure S1). Then, the *C. chinensis* adults were fed with mung beans containing CS-*siHIF1α* nanoparticles and then subjected to hypoxic treatment. Insects treated with CS-*siGFP* were set as the control. After 48 h of hypoxia, the expression of HIF1α at both transcript and protein levels was assessed by RT-qPCR and Western blotting, respectively. The results showed a significant downregulation of *HIF1α* expression, with decreases of 51.2% at the transcript (mRNA) level and 64.8% at the protein level (Figure 3A,B). Kaplan–Meier survival analysis revealed that *HIF1α* knockdown significantly reduced the survival of *C. chinensis* adults under hypoxia compared with the results for the CS-*siGFP* control group (log-rank test, *p* < 0.0001; Figure 3C). The median survival time of the CS-*siHIF1α* group was 72 h, whereas that of the control group was 168 h. *HIF1α* expression was monitored over 168 h of hypoxic exposure. Levels were significantly lower in the CS-*siHIF1α* group compared to the CS-*siGFP* control group from 24 to120 h, but no significant differences were observed at the onset (0 h) or toward the end of the hypoxic treatment (144 h and 168 h) (Figure 3D).

### 3.3. Effect of HIF1α on Antioxidative Response of C. chinensis Adults Under Hypoxia

**Figure 4:**
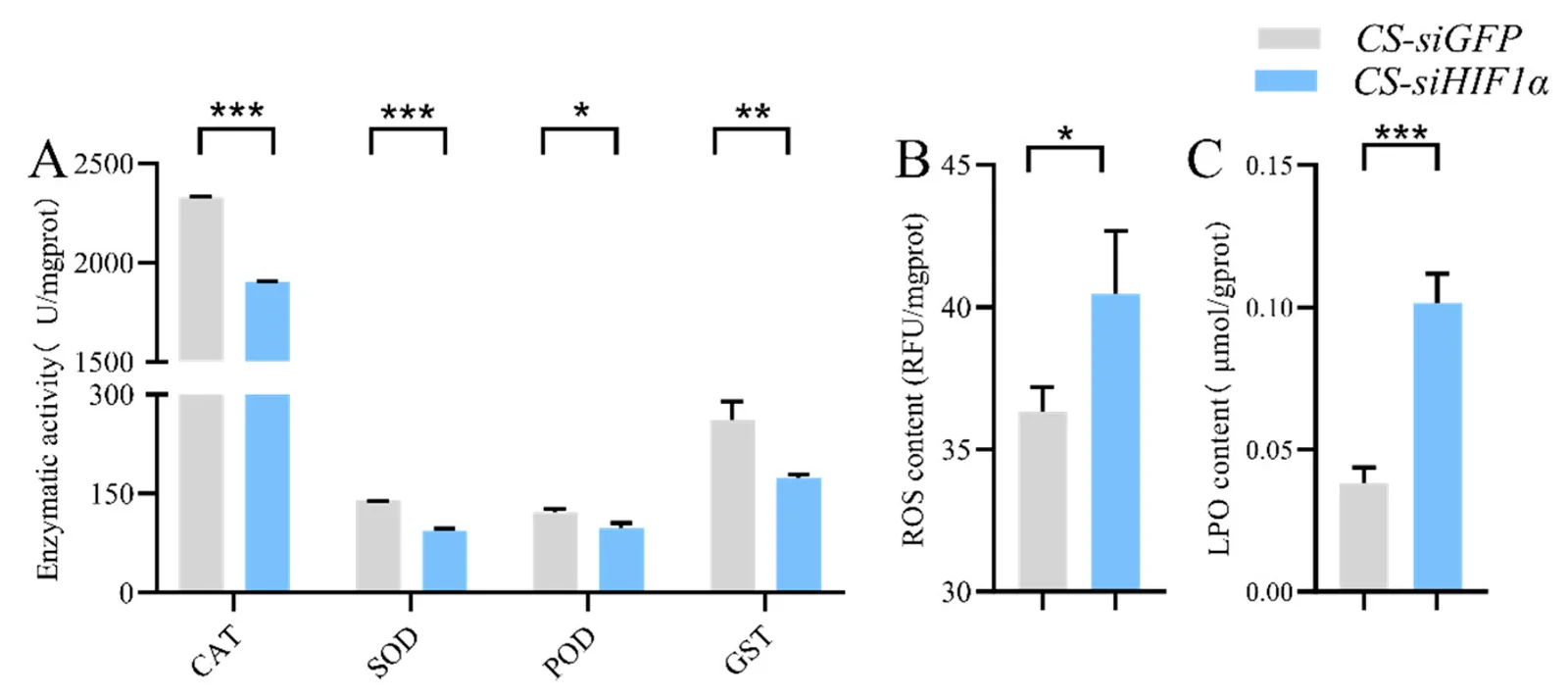
Effect of *HIF1α* on the oxidative stress response of *C. chinensis* adults under hypoxia. (**A**) Characteristic antioxidant enzyme activity. (**B**) ROS and (**C**) LPO content. The significant differences are denoted with * *p* < 0.05, ** *p* < 0.01, and *** *p* < 0.001 using an independent *t*-test. The bars represent mean ± SE of three replicates. CAT: catalase, SOD: superoxide dismutase, POD: peroxidase, GST: glutathione S-transferase, ROS: reactive oxygen species, and LPO: lipid hydroperoxide.

To investigate the role of *HIF1α* in oxidative stress responses, the changes in the activities of four antioxidant enzymes (catalase (CAT), superoxide dismutase (SOD), peroxidase (POD), and glutathione S-transferase (GST)) and the contents of two peroxides (reactive oxygen species (ROS) and lipid hydroperoxide (LPO)) in *C. chinensis* adults after CS-*siHIF1α* treatment under hypoxia were examined (Figure 4). The insects treated with CS-*siGFP* were set as the control (Figure 4). The activities of antioxidant enzymes, including CAT, SOD, POD and GST in adult insects in the CS-*siHIF1α* group were significantly decreased compared to the levels for the controls. Among of these, the activities of CAT and SOD was decreased by 18.3% and 32.1%, respectively (Figure 4A). Meanwhile, the levels of ROS and LPO were significantly increased in the CS-*siHIF1α* group, by 10.2% and 62.3%, respectively, compared to the levels for the controls (Figure 4B,C).

### 3.4. Comprehensive Lipids Profiling of C. chinensis Adults Under Hypoxia

**Figure 5:**
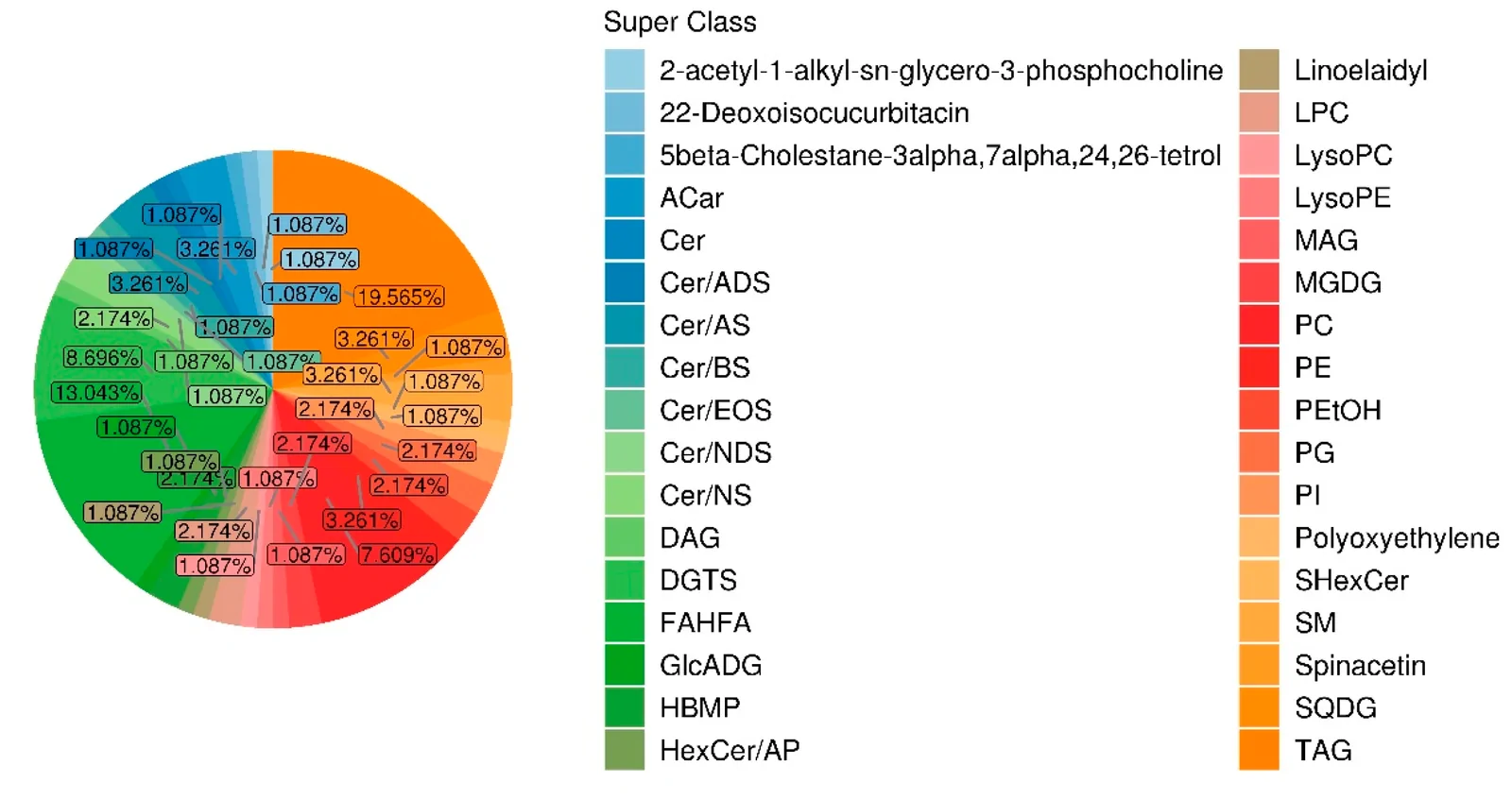
Lipid species in *C. chinensis* adults under hypoxia. ACar: acylcarnitine, Cer: ceramides, Cer-ADS: ceramide alpha-hydroxy fatty acid-dihydrosphingosine, Cer-AS: ceramide alpha-hydroxy fatty acid-sphingosine, Cer-BS: ceramide beta-hydroxy fatty acid-sphingosine, Cer-EOS: ceramide esterified omega-hydroxy fatty acid-sphingosine, Cer-NDS: ceramide non-hydroxyfatty acid-dihydrosphingosine, Cer-NS: ceramide non-hydroxyfatty acid-sphingosine, DAG: diacylglycerol, DGTS: diacylglyceryl trimethylhomoserine, FAHFA: fatty acid ester of hydroxyl fatty acid, GlcADG: gcuronosyldiacylglycerol, HBMP: hemibismonoacylglycerophosphate, HexCer-AP: hexosylceramide alpha-hydroxy fatty acid-phytospingosine, LPC: lysophophatidylcholine, LysoPC: lysophophatidylcholine, LysoPE: lysophosphatidylethanolamine, MAG: monoacylglycerol, MGDG: monogalactosyldiacylglycerol, PC: phosphatidylcholine, PE: phosphatidylethanolamine, PEtOH: phosphatidylethanol, PG: phosphatidylglycerol, PI: phosphatidylinositol, SHexCer: sulfurHexosylceramide hydroxyfatty acid, SM: sphingomyelin, SQDG: sulfoquinovosyl diacylglycerol, and TAG: tacylglycerol.

A non-target lipidomics analysis was firstly performed to investigate the changes in lipid metabolism of *C. chinensis* adults. A total of 1867 lipid molecular species in *C. chinensis* were separated and characterized in positive- and negative-ion modes. These lipids were assigned to 46 subclasses, among which six major lipids were triacylglycerol (TAG, 17.01%), phosphatidylcholine (PC, 10.78%), ceramide species (Cer, 9.69%), diacylglyceryltrimethylhomo-serine (DGTS, 7.05%), acylglucuronosyldiacylglycerol (AcylGlcADG, 6.49%), hemibismonoacylglycerophosphate (HBMP, 6.11%), and sulfoquinovosyl diacylglycerol (SQDG, 4.51%) (Figure 5). Among these, TAG was the most abundant type of lipid; the key TAG species identified were TAG (16:1/18:1/18:1), followed by TAG (18:1/18:1/18:3), TAG (16:0/18:1/18:3) and TAG (13:0/20:0/20:0). Moreover, the content of Cers was also high, including ceramide alpha-hydroxy fatty acid-phytospingosine (Cer/AP) Cer/AP (t25:2/12:0), followed by Cer/AP (t20:0/15:1), and hexosylceramide non-hydroxyfatty acid-sphingosine (HexCer/NS) HexCer/NS (d25:3/18:2). For the PCs, the content of PC (18:2/18:2) was the highest, followed by PC (18:1/18:1) and PC (18:1/18:2).

### 3.5. Effect of CcHIF1α on Sphingolipid Metabolism Changes Under Hypoxia

**Figure 6:**
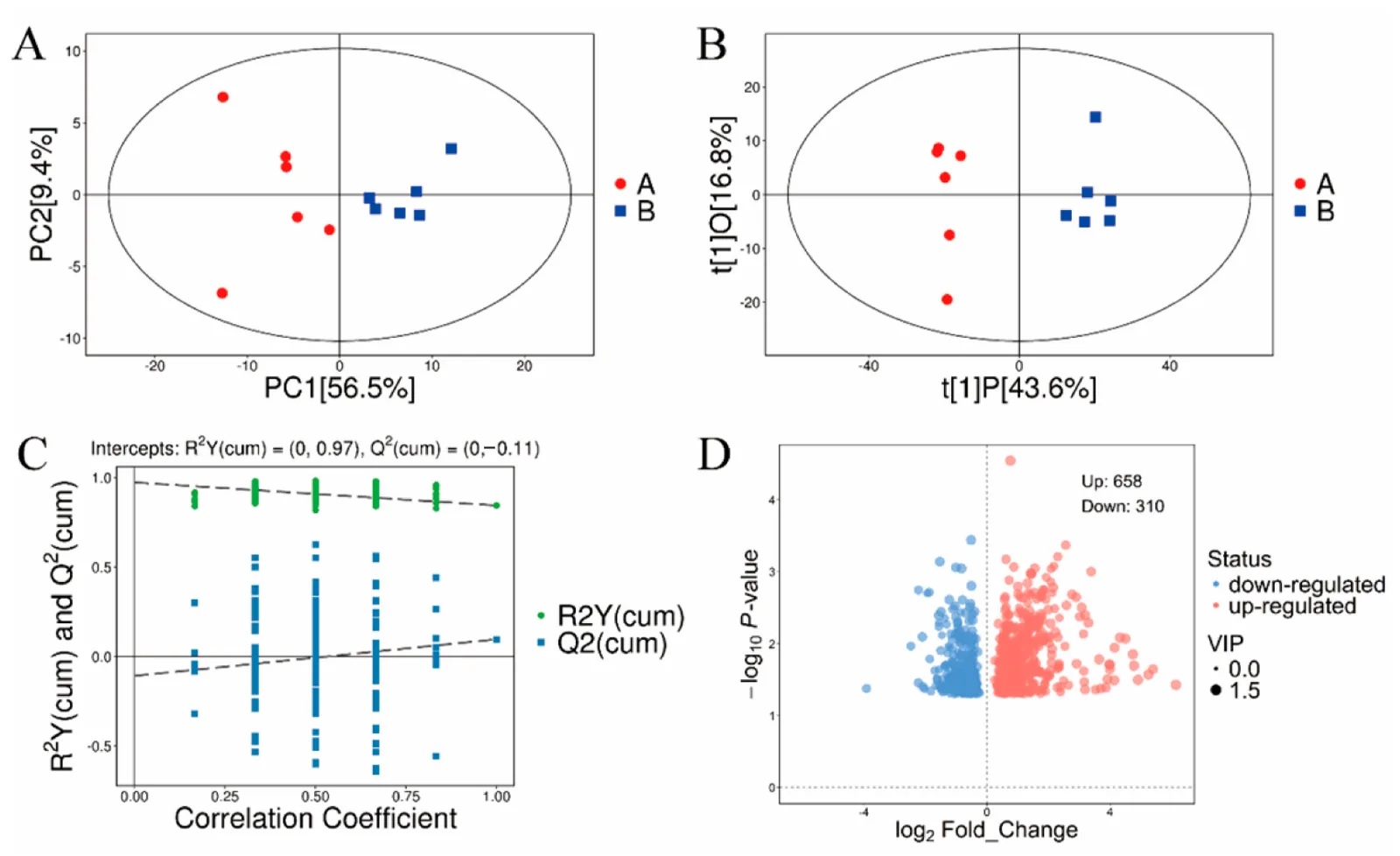
Multivariate statistical analysis of lipidomics in *C. chinensis* adults under hypoxia. (**A**) PCA score plot, (**B**) OPLS-DA score plots, (**C**) the permutation test for validating the OPLS-DA model, and (**D**) volcano plot of differentially expressed lipids (DELs) between the CS-*siHIF1α* and CS-*siGFP* groups. Group A: CS-*siGFP*; Group B: CS-*siHIF1α*.

The differences in lipidomic profiles between the CS-*siHIF1α* and CS-*siGPF* groups were analysed by principal component analysis (PCA) and orthogonal partial least squares-discriminant analysis (OPLS-DA) models. PCA models showed that components 1 and 2 represented 56.5% and 9.4% of total lipids of the samples, respectively (Figure 6A). The substances from the two groups (CS-*siHIF1α* and CS-*siGPF*) were well separated in this model, revealing that the lipids were significantly different. The predictability parameters of R^2^Y = 0.839 and Q^2^ = 0.082, respectively, were noted. OPLS-DA score plots further showed a clear separation between the siHIF1α and siGPF groups (Figure 6B). The OPLS-DA model was validated by permutation testing, yielding R^2^Y = 0.97 and Q^2^ = −0.11 (Figure 6C), and showed good model performance. Compared to the CS-*siGPF* group, a total of 968 differentially expressed lipids (DELs) (658 upregulated and 310 downregulated DELs) were obtained in the CS-*siHIF1α* group, which are shown in the volcano diagram (Figure 6D).

**Figure 7:**
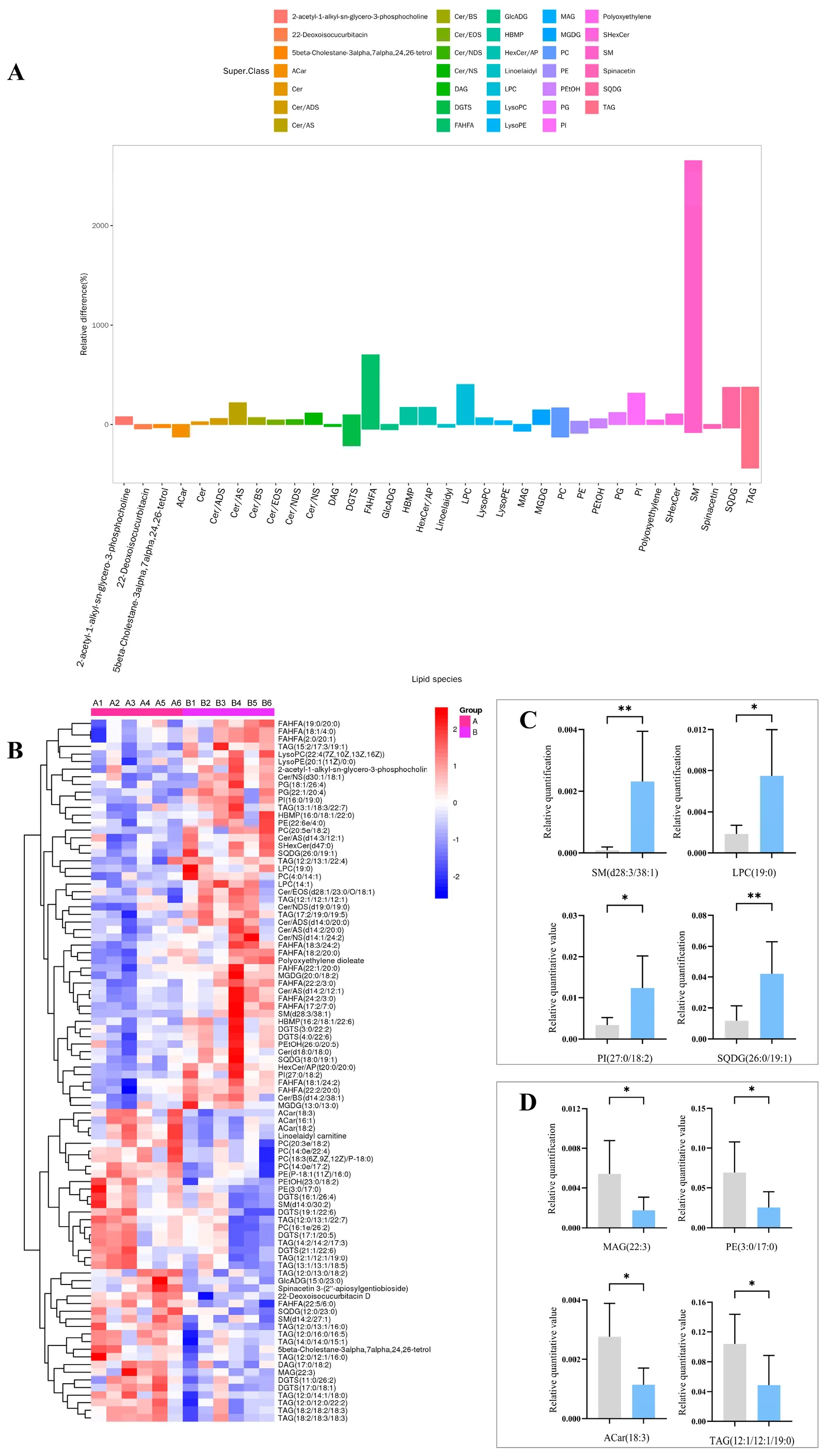
Characteristic lipids significantly changed in *C. chinensis* adults due to CS-*siHIF1α* treatment under hypoxia. (**A**) Bar plot from the CS-*siHIF1α* group and the CS-*siGFP* group. Each bar represents a class of lipids. The vertical axis denotes the relative percentage change in content compared to that of the control, while the horizontal axis indicates the lipid classification. A zero value denotes equal content in both groups; a positive value signifies higher content in the CS-*siHIF1α* group, and a negative value indicates higher content in the CS-*siGFP* group. (**B**) Heat map analysis. Relative quantification of the top four upregulated DELs (**C**), and top four downregulated DELs (**D**). Metabolite levels that were elevated are shown in red, and those that were reduced in blue. The significant differences are denoted with * *p* < 0.05, ** *p* < 0.01 using independent *t*-test. Each rectangle represents the Pearson correlation coefficient between the metabolite in the row with a sample in the column. This was calculated by averaging the normalized peak areas, with z-score scaling of the controls. Each experiment was performed six times.

Notably, 92 lipids (including 51 upregulated and 41 downregulated DELs) were accurately identified. These DELs were categorized into 34 lipid subclasses, with the magnitude of change for each subclass visually displayed in a bar chart (Figure 7A). A larger absolute value of the relative change signifies a greater disparity between the two groups. Among these lipid subclasses, 3 DELs (1 upregulated, 2 downregulated) were identified as sphingomyelin (SM), the positive value of relative change was 2652.96%, and the negative value was −81.18%, revealing the higher content in the CS-*siHIF1α* group. Fatty acid esters of hydroxy fatty acids (FAHFA) with 12 DELs (2 upregulated, 1 downregulated) exhibited a positive value of relative change of 699.81%, while the negative value was −47.23%; triacylglycerol (TAG) subclasses with 18 DELs (5 upregulated, 13 downregulated) showed a positive value of relative change of 375.98%, while the negative value was −437.25%; diacylglyceryltrimethylhomoserine (DGTS) with 8 DELs (2 upregulated, 6 downregulated) showed a positive value of relative change of 95.89%, while the negative value was −212.73%; lysophosphatidylcholine (LPC) with 2 DELs (2 upregulated) exhibited a positive value of relative change of 401.76%. Specifically, all DELs within a subclass (i.e., Cer-related, Cer (d18:0/18:0), Cer/AS (d14:2/12:1) and Cer/BS (d14:2/38:1)) were increased in the CS-*siHIF1α* group. The details are presented in Table S7.

**Figure 8:**
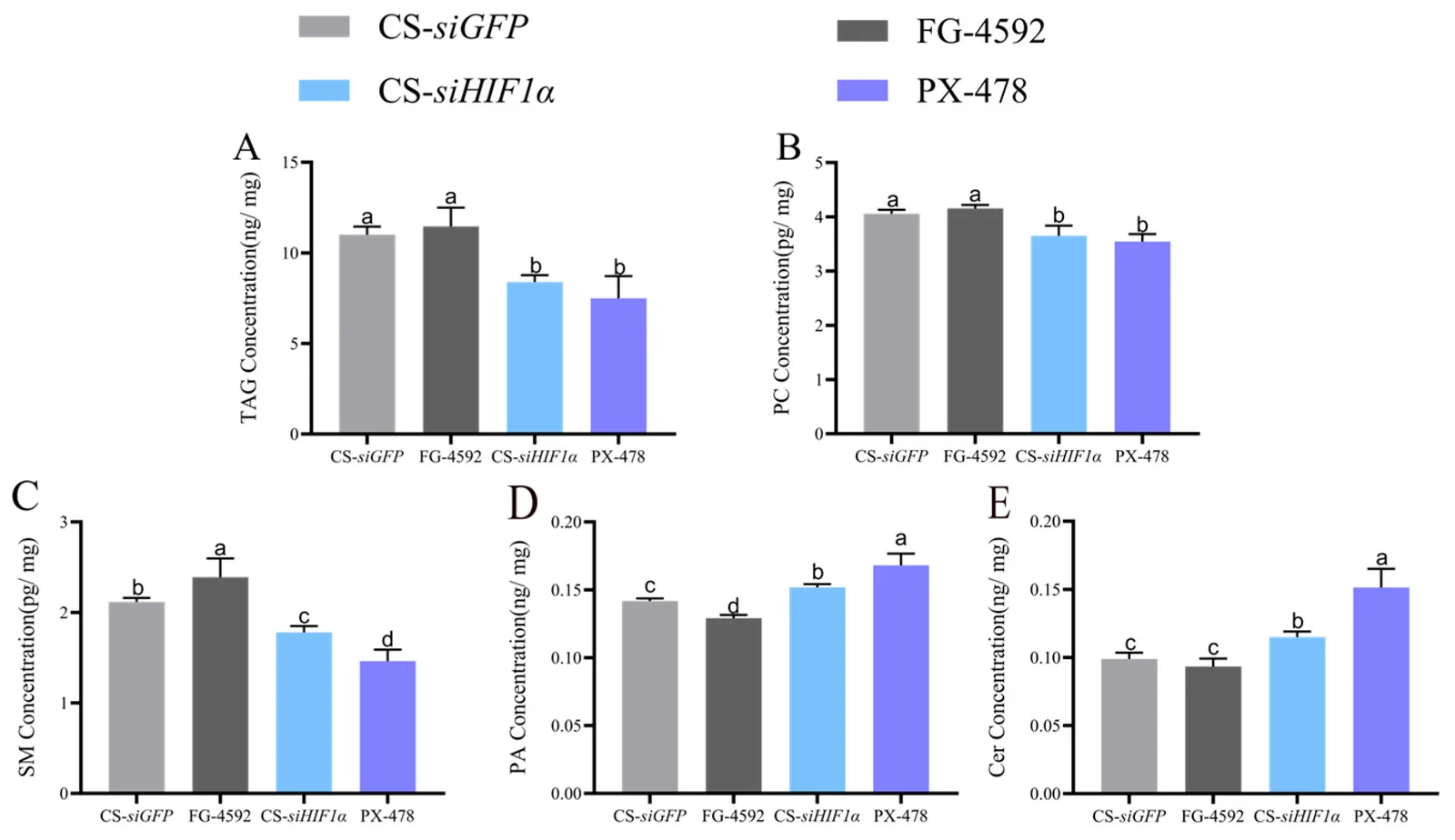
Changes in characteristic lipid content in *C. chinensis* adults under hypoxia. Content level of (**A**) TAG, (**B**) PC, (**C**) SM, (**D**) PA, and (**E**) Cer in different groups (CS-*siGFP*, CS-*siHIF1α*, PX-478, or FG-4592). Values followed by the different small letters were significantly different according to Tukey’s test (*p* < 0.05). The bars represent mean ± SE of three repeats. PX-478, *HIF1α* inhibitor; FG-4592, HIF activator; TGA, triglyceride; PC, phosphatidylcholine; SM, sphingomyelin; PA, palmitic acid; Cer, ceramide contents.

Their content changes are clearly visualized in a heatmap (Figure 7B). The detailed information is shown in Table S7. It shows that the increase in SM (d28:3/38:1) (27.52-fold), LPC (19:0) (4.07-fold), PI (27:0/18:2) (3.65-fold) and SQDG (26:0/19:1) (3.58-fold), were larger than those of the other upregulated DELs (Figure 7C), while the decreases in MAG (22:3) (0.32-fold), PE (3:0/17:0) (0.36-fold), ACar (18:3) (0.42-fold) and TAG (12:1/12:1/19:0) (0.47-fold), were more significantly than those for the other downregulated DELs (Figure 8D). Among these DELs, most lipids were related to sphingolipid metabolism, including SM (d28:3/38:1) (27.52-fold), Cer (d18:0/18:0) (1.26-fold), Cer/ADS (d14:0/20:0) (1.63-fold), Cer/BS (d14:2/38:1) (1.68-fold), Cer/AS (d14:2/12:1) (2.38-fold) and HexCer/AP (t20:0/20:0) (2.72-fold) and were significantly increased.

The correlations among these 92 DELs were assessed using a Pearson correlation heatmap (Figure S2). It directly presents the potential regulation relationships among these metabolites in insects due to *HIF1α* exposure under hypoxia. A positive correlation occurs when the change trends of two metabolites align, whereas a negative correlation is identified when their trends diverge. The correlation coefficient, R, quantifies this relationship, where a larger absolute value signifies a stronger correlation and a smaller value a weaker one. For example, Cer (d18:0/18:0) showed a positive correlation with Cer/ADS (d14:0/20:0) and FAHFA (17:2/7:0) (r = 0.93 and r = 0.73, respectively); Cer (d18:0/18:0) exhibited a negative correlation with SM (d14:0/30:2) and TAG (12:1/12:1/19:0) (r = −0.42 and r = −0.58, respectively).

A total of 92 DELs were enriched into the KEGG database to elucidate the impact of *HIF1α* on metabolic pathways. Manual searching of the DELs in the KEGG database showed that glycerophospholipid metabolism (23 hits), glycerolipid metabolism (22 hits), sphingolipid metabolism (13 hits), thermogenesis pathway (3 hits), and ether lipid metabolism (1 hit) were disturbed (Table S7).

### 3.6. Effect of HIF1α on Characteristic Lipids Synthesis in C. chinensis Adults Under Hypoxia

After 48 h of hypoxia exposure following treatment with CS-*siGFP*, CS-*siHIF1α*, PX-478 (*HIF1α* inhibitor), or FG-4592 (HIF activator), the contents of characteristic lipids, including triglyceride (TAG), phosphatidylcholine (PC) sphingomyelin (SM), palmitic acid (PA), and ceramide (Cer), in *C. chinensis* adults showed group-specific alterations (Figure 8).

Under hypoxic conditions, the changes in TAG and PC content followed a similar pattern across the four treatment groups. The PX-478 and CS-*siHIF1α* groups exhibited significantly lower levels of both lipids compared to those in the FG-4592 and CS-*siGFP* groups, with average values of 11.23 ± 0.75 and 7.93 ± 0.95 ng/mg, respectively, and there was no significant difference between the PX478 and CS-*siHIF1α* groups, nor between the FG-4592 and CS-*siGFP* groups (Figure 8A,B). SM levels varied across the groups (*p* < 0.05). The highest content was observed in the FG-4592 group (2.39 ± 0.21 ng/mg), followed by the CS-*siHIF1α* group (2.12 ± 0.05 ng/mg), the CS-*siGFP* group (1.78 ± 0.07 ng/mg), and the PX-478 group (1.46 ± 0.13 ng/mg) (Figure 8C). PA content also showed significant variation (*p* < 0.05), following an inverse pattern across groups. Levels were ranked as follows, from high to low: PX-478 > CS *siHIF1α* > FG-4592 > CS *siGFP* (Figure 8D). Similar to PA, Cer content was highest in the PX-478 group (0.15 ± 0.01 ng/mg), followed by the CS-*siHIF1α* group (0.12 ± 0.00 ng/mg), and lowest in the FG-4592 (0.10 ± 0.00 ng/mg) and CS-*siGFP* (0.09 ± 0.00 ng/mg) groups, with no significant difference between the latter two (Figure 8E).

### 3.7. Effect of CcHIF1α on the Expression of Genes Involved in Sphingolipid Metabolism Under Hypoxia

**Figure 9:**
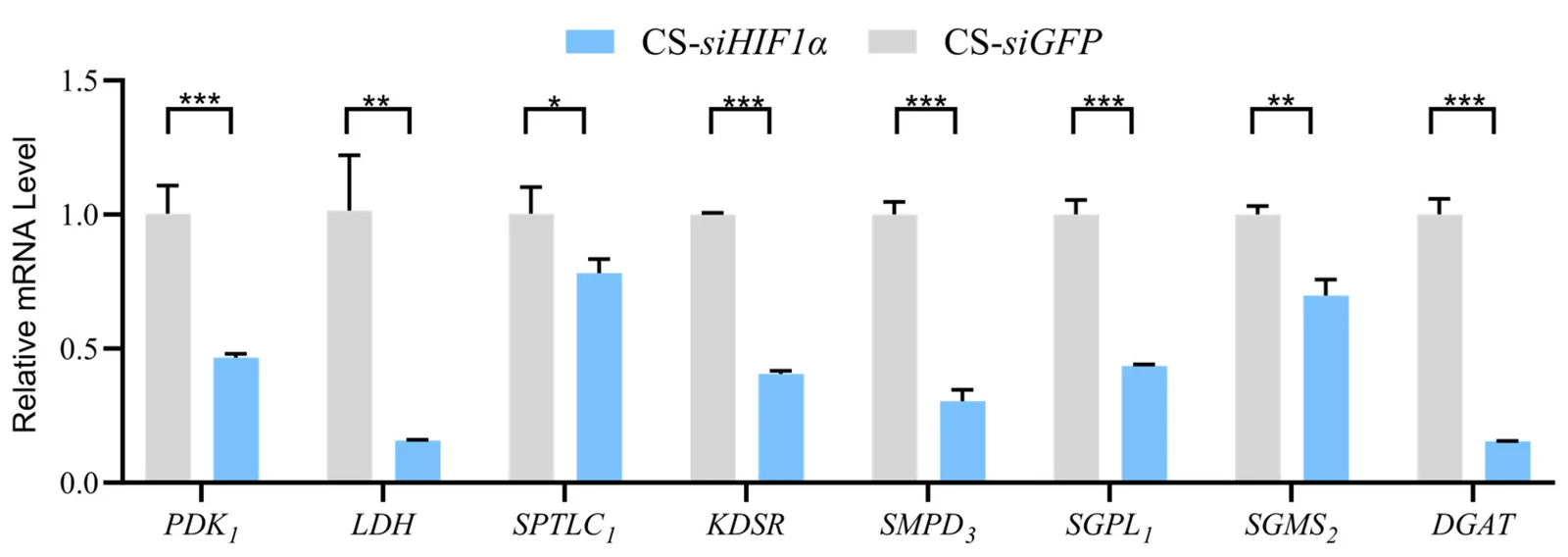
Partial genes related to lipid metabolism were changed after knockdown of *HIF1α*. The significant differences are denoted with * *p* < 0.05, ** *p* < 0.01, and *** *p* < 0.001 using independent *t*-test. The bars represent mean ± SE of three repeats. *PDK_1_*: pyruvate dehydrogenase kinase, *LDH*: lactate dehydrogenase, *SPTLC_1_*: serine palmitoyl transferase 1, *KDSR*: 3-Ketodihydrosphingosine Reductase, *SMPD_3_*: sphingomyelin phosphodiesterase, *SGPL_1_*: sphingosine-1-phosphate lyase 1, *SGMS_2_*: sphingomyelin synthase 2, and *DGAT*: diacylglycerol acyltransferase.

The changes in expressions of eight genes in *C. chinensis* adults after *HIF1α* knockdown under hypoxia were further analysed using RT-qPCR. These genes encode lactate dehydrogenase (*LDH*), pyruvate dehydrogenase kinase 1 (*PDK_1_)*, serine palmitoyl transferase 1 (*SPTLC_1_*), 3-ketodihydrosphingosine Reductase (*KDSR*), sphingomyelin phosphodiesterase (*SMPD_3_*), sphingosine-1-phosphate lyase 1 (*SGPL_1_*), sphingomyelin synthase 2 (*SGMS_2_*) and diacylglycerol acyltransferase (*DGAT*). Following *HIF1α* knockdown (CS-*siHIF1α*), the mRNA levels of these genes, especially *PDK_1_* (0.46-fold), *KDSR* (0.41-fold), *SMPD_3_* (0.30-fold), *SGPL_1_* (0.44-fold), and *DGAT* (0.15-fold), were significantly decreased compared to the levels of the controls (CS-*siGFP*) (Figure 9).

## 4. Discussion

Modified atmospheres (MAs) based on low oxygen have been widely used for insect pest control in grain storage, while the stored-product insects, especially weevils, which live inside of the grain seed during the process of growth and development, show highly hypoxia-adaptive capability. As a key mediator of hypoxia adaptation in insects, *HIF1α* may represent a potential target for novel insecticides and pest control methods. However, the details of the mechanism of *HIF1α* in regulating the insect hypoxia adaption are still not clearly understood. In this study, following the whole sequence of *HIF1α* of *C. chinensis* was obtained using the Race PCR technique. RNA interference was performed using synthesized siRNA targeting the *HIF1α*. The potential role of *CcHIF1α* in hypoxic adaptation and lipid metabolism of *C. chinensis* under low-oxygen conditions was then investigated. The findings could further expand our understanding of *HIF1α* function and may provide new insights for informing the development of hypoxia-based pest control strategies.

As previously reported, the *CcHIF1α* subunit shared high sequence similarity with its homologues from *H. sapiens*, *D. melanogaster*, *C. elegans* and *T. castaneum,* and all contained the conserved domains (i.e., bHLH, PAS, and C-TAD). bHLH-PAS domains in *HIF1α* function as transcription factors and coactivators for transcriptional regulation in bacteria, archaea, and eukaryote, eliciting responses to environmental and intracellular changes in oxygen, light, redox potential, small ligands, and overall cellular energy levels. The PAC domain connects with the PAS domain and functions as a trans-activation domain at the C-terminus of *HIF1α* [32]. The oxygen-dependent degradation (ODD) domain and the C-TAD domain function as a transactivation domain that modulates the stability of *CcHIF1α* as a function of oxygen availability [32,33]. The sequence alignment further showed that *CcHIF1α* was highly conserved from *C. chinensis* and another Coleopteran. There was a high homology of amino acid sequences between *C. chinensis* and other Coleoptera insects, especially *C*. *maculatus* (Figure 2 and Table S4). Under hypoxic conditions, insects would die due to insufficient energy supply and severe cellular damage induced by high oxidative stress. The mortality of adult *C. chinensis* increased with prolonged hypoxic exposure and reached 100% by day 11. This effect was significantly more pronounced following *HIF1α* knockdown. It was similar to the results of a previous report regarding *Aleuroglyphus ovatus* under hypoxic conditions, which directly showed the important role of *HIF1α* for the survival of insects under hypoxia [34].

Metabolic reprogramming is an important adaptive mechanism that cells undergo to survive and function under hypoxic conditions. *HIF1α* is considered to be involved in metabolic shifting to glycolysis rather than in oxidative metabolism via regulating the function of genes involved in gluconeogenesis and oxidative stress [35]. Under hypoxia, the levels of ROS and LPO, two important indicators for oxidative stress, were significantly increased in *C. chinensis* adults after *HIF1α* knockdown. ROS is generated during hypoxia by mitochondrial complex III; its production increases exponentially in cells under 5% oxygen [36]. Excessive ROS production usually reveals the inadequate antioxidant defences, and it might damage cell structures, including proteins, DNA, and lipids, finally contributing to cell death [37]. However, under hypoxic conditions, mitochondrial ROS may induce the inactivation of PHDs, thereby promoting the expression of HIF1α proteins [38]. Furthermore, ROS have been suggested to be involved in regulating insect diapause through either insulin signalling or the HIF/miR-34/ACS-PK pathway. These processes collectively lead to the suspension of all developmental stages and extend insect lifespan [38,39,40]. However, the relationship between ROS and hypoxia signalling is still not clear.

Correspondingly, the activities of antioxidant enzymes (CAT, SOD, POD, and GST)—key components of the antioxidant defence system—were reduced following *HIF1α* knockdown in *C. chinensis* adults under hypoxic conditions. Lipid hydroperoxide (LPO) is an inert biomarker and an active mediator of damage through its ability to form covalent adducts with proteins and DNA, altering function and triggering secondary responses [37,41,42]. Our results further suggest that *HIF1α* may play a protective role against oxidative stress damage under hypoxic conditions and may be associated with reducing ROS and LPO levels. However, the underlying mechanisms need to be further studied.

Elevated oxidative stress can induce lipid peroxidation, which may contribute to alterations in lipid metabolism [43,44]. The details of changes were further investigated using lipidomic methods. Due to *CcHIF1α* knockdown, lipid metabolism significantly changed. A total of 92 differentially expressed lipids (DELs) belonging to 34 subclasses, such as TAGs, FAHFA, DGTSs, Cers, and PC, were identified. These findings further highlight the important role of *HIF1α* in regulating lipid metabolism, particularly sphingolipid metabolism.

PC content was significantly decreased in adult *C. chinensis* following *HIF1α* knockdown under hypoxic conditions. In contrast, LPC—an important metabolite derived from PC via the action of enzymes such as phospholipase A2 in the glycerophospholipid metabolism pathway—was found to be increased. This might accelerate cell apoptosis and inflammatory damage under hypoxic stress, resulting in insect death [45]. As a critical precursor for both α-linolenic acid (α-LA) and arachidonic acid (AA), PC supports diverse biological processes essential for insect development and survival. Metabolites derived from LA are crucial for hormone synthesis and development, while AA serves as a key signalling lipid mediating inflammation, cellular signalling, and stress responses [46,47]. Previous studies have reported that arachidonic acid metabolism is associated with specific phenotypic variations following acute hypoxia exposure, and *HIF1α* may regulate this pathway by activating cytochrome c oxidase (COX) [48]. Furthermore, the observed decrease in PC suggests an additional mechanism by which *HIF1α* might influence AA metabolism through reduced availability of its PC-derived precursor.

Additionally, many lipids associated with sphingolipid metabolism—including TAG, SM, Cer, PC and FAHFA species—were significantly altered in *C. chinensis* adults following *HIF1α* knockdown under hypoxia. TAGs are the main principal neutral lipids in insects, which are essential for their growth, development, and reproduction, protecting them from the effects of pathogens, oxidative stress, and other stress harm [43,49]. Sphingomyelin (SM), the predominant sphingolipid in cellular membranes, is synthesized from ceramide (Cer) by sphingomyelin synthase (SMS) and can be hydrolysed back to Cer by sphingomyelinases (SMases) in response to cellular demand; ceramides (Cers) are recognized as key stress-signalling mediators that integrate diverse stress signals to influence cell fate, primarily by arresting growth and promoting the death of damaged or potentially harmful cells [50,51]. In this study, following *HIF1α* knockdown under hypoxic conditions, the levels of SM (d14:0/30:2) and SM (d14:2/27:1) were significantly decreased in insects, along with several downstream ceramide species, including Cer (d18:0/18:0), Cer/ADS (d14:0/20:0), Cer/BS (d14:2/38:1), Cer/AS (d14:2/12:1), and HexCer/AP (t20:0/20:0). The levels of Cer species and their balance with other sphingolipids are critically important for insect survival. It has been reported that homocysteine-induced insulin resistance was ameliorated after adipocyte-specific *HIF1α* knockout, while the accumulation of ceramide dose could enhance insulin resistance [21,52,53]. In addition, increased ROS levels can induce ceramide accumulation by activating related enzymes such as SMase and ceramide synthase [54]. The relative levels of some phosphatidylcholines, such as PC (14:0e/17:2), PC (16:1e/26:2), PC (18:3 (6Z,9Z,12Z)/P-18:0), PC (20:3e/18:2) and PC (14:0e/22:4), were also decreased after *HIF1α* knockdown. This decrease may promote sphingomyelin synthesis via SMS, which transfers the phosphocholine headgroup from PC to ceramide [55]. However, the mechanism of *HIF1α* in regulating ceramide generation still requires further investigation.

A complementary validation was performed using the PX-478 and FG-4592 groups to analyse the characteristic lipids (TAG, PC, SM, Cer and PA). The PX-478 group exhibited behaviour consistent with the *HIF1α* knockdown group, whereas the FG-4592 group exhibited opposite behaviours to those observed following *HIF1α* knockdown.

Specifically, following *HIF1α* knockdown, TAG, PC, and SM levels were markedly reduced, whereas Cer and PA content was significantly increased. TAG, PC, and SM levels decrease, which significantly inhibits insect reproduction and disrupts normal growth and development [56,57,58]. In *Nilaparvata lugens*, an increase in ceramide levels disrupts the insect reproductive system [44]. PA is a saturated fatty acid. Its excessive accumulation causes lipotoxicity, which affects reproductive development [59,60]. The results suggest a potential role of *HIF1α* in regulating lipid metabolism, particularly in sphingolipid metabolism remodelling in insects under hypoxia.

Correspondingly, the expression of these metabolically related genes was also altered. The expression levels of *PDK_1_* and *LDH*, two critical enzymes involved in both oxidative phosphorylation and glycolysis, were significantly decreased in *C. chinensis* adults after *HIF1α* knockdown under hypoxia. Previous studies have reported that the promoter regions of both *PDK_1_* and *LDH* contain hypoxia response elements (HREs) and are directly regulated by *HIF1α* [61,62]. The reduction in their transcription levels may contribute to the inhibition of the conversion of glucose to pyruvate and lactate, potentially resulting in inadequate energy, the accumulation of ROS, and lipid synthesis blocking [63]. It has been reported that *PDK_1_* expression increases in hypoxia in a *HIF1α*-dependent manner, which not only affects the glycolytic reprogramming under hypoxia but also controls triacylglycerol hydrolysis [64,65]. As we know, ceramide synthesis occurs mainly from three pathways; it starts with saturated fatty acids and palmitate intake, activating the de novo synthesis of ceramide. Notably, the content of palmitic acid was increased in *C. chinensis* adults after *HIF1α* knockdown, and the expression of two genes encoding rate-limiting enzymes, *SPTLC1* and *KDSR* in the pathway of de novo ceramide biosynthesis, were downregulated, possibly leading to the accumulation of palmitic acid and the inhibition of ceramide. In the sphingomyelinase and salvage pathways, both sphingomyelin and its downstream metabolite phosphatidylcholine were decreased. Moreover, the expression of *SMPD3*—which encodes sphingomyelin phosphodiesterase, a key rate-limiting enzyme—was significantly downregulated following *HIF1α* knockdown, potentially contributing to the observed reduction in ceramide levels. Previous studies have also demonstrated that *HIF1α* accumulation in mouse adipose tissue increases ceramide levels by activating *SMPD3*, thereby accelerating the conversion of SM to Cer [20].

**Figure 10:**
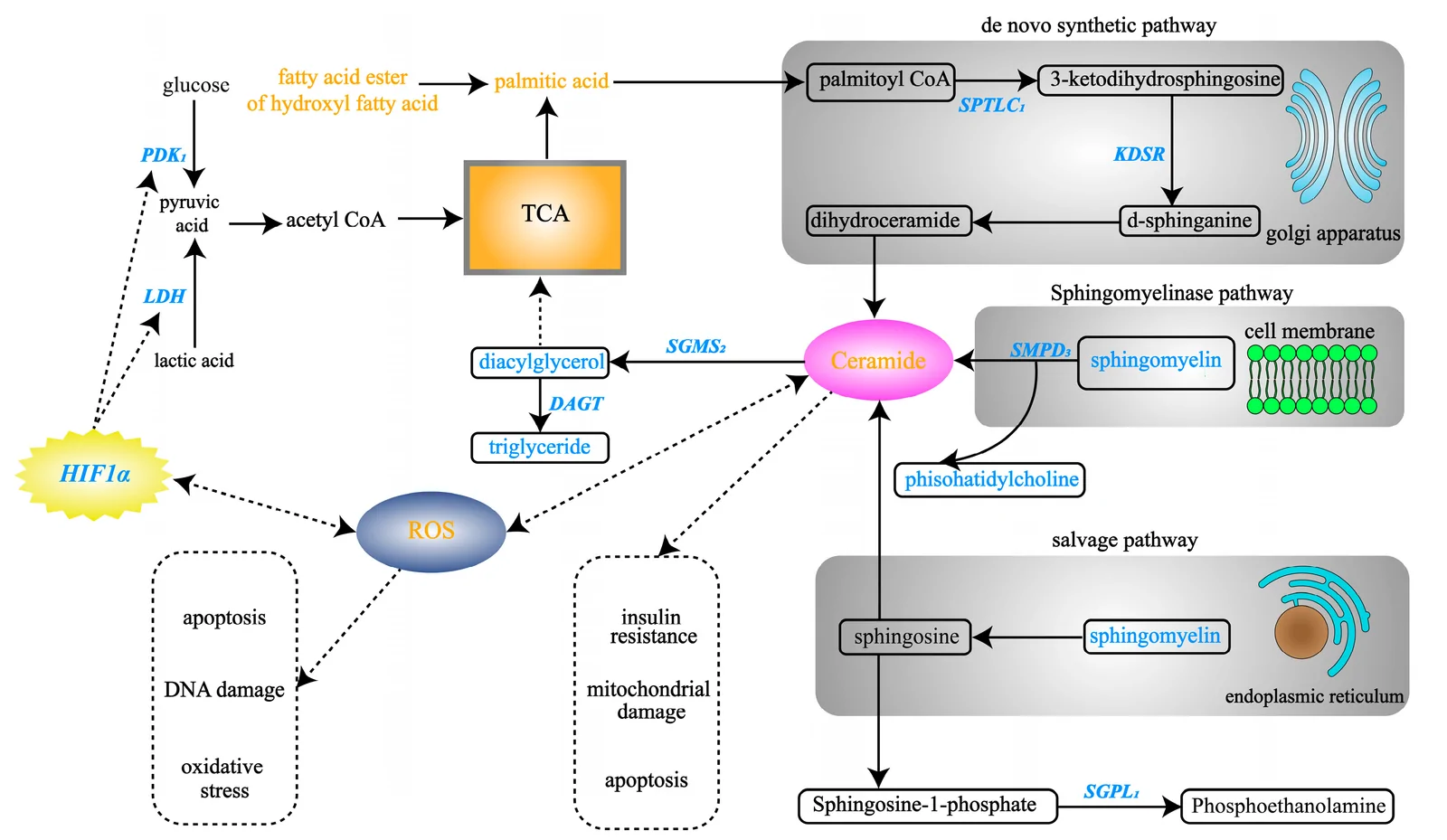
Schematic diagram of sphingolipid metabolism in *C. chinensis* after knockdown of *HIF1α*. Orange font indicates upregulated substance content or gene expression, while blue font represents downregulated levels. *PDK_1_*: pyruvate dehydrogenase kinase, *LDH*: lactate dehydrogenase, *SPTLC_1_*: serine palmitoyl transferase 1, *KDSR*: 3-Ketodihydrosphingosine Reductase, *SMPD_3_*: sphingomyelin phosphodiesterase 3, *SGPL_1_*: sphingosine-1-phosphate lyase 1, *SGMS_2_*: sphingomyelin synthase 2, and *DGAT*: diacylglycerol acyltransferase.

However, the total ceramide content was significantly increased in *C. chinensis* adults after *HIF1α* knockdown under hypoxia. The results suggest that there might be other pathways activated for ceramide generation. Therefore, the pathway of ceramide catabolism was also examined. Here, the genes *SMGS2* and *DAGT*, encoding key enzymes catalysing the synthesis of DAG and TAG using ceramide and phosphatidylcholine as substrates, were both found to be downregulated following *HIF1α* knockdown. This downregulation led to a marked accumulation of total ceramide, particularly the C18-sphingosine species. Accordingly, the levels of downstream metabolites—including DAG and TAG—were found to be decreased, further supporting the accumulation of ceramide. Thus, the inhibition of ceramide catabolism may be the primary cause of ceramide accumulation in adult *C. chinensis* following *HIF1α* knockdown under hypoxic conditions. The results of lipidomic analysis provided the changes in these characteristic sphingolipids (Figure 8). The accumulation of ceramide could further accelerate insulin resistance, oxidative stress, inflammation, and cell death [66,67,68]. It has been reported that ceramides containing C16:0 and C18:0 are particularly detrimental. Among these, three specific species (C16:0, C18:0, and C24:1) have been more strongly associated with coronary artery disease and metabolic abnormalities such as insulin resistance [23]. These results reveal a protective role of *HIF1α* against the adverse effects of oxidative stress and specific ceramide species in insects under hypoxic stress. A schematic diagram of the proposed relationships is presented in Figure 10.

In this study, the full sequence of *HIF1α* of *C. chinensis* was obtained, and its function in regulating lipid metabolism was further studied. As previously reported, *CcHIF1α* also showed highly conserved structural domains and functions. Under hypoxic conditions, after *HIF1α* knockdown, *C. chinensis* adults showed higher mortality than that of those in the CS-*siGFP* groups. Meanwhile, oxidative stress was elevated, and lipid metabolism was reprogrammed due to *HIF1α* knockdown. Specifically, the content of ROS and ceramide species was increased, and the downregulation of the expression of key genes (*SPTLC_1_*, *KDSR*, *SMPD_3_*, *SGPL_1_*, and *SGMS_2_*) may be associated with the increased content of these compounds. However, the relationship between this regulation and ROS and ceramides requires further study in the future. Thus, *HIF1α* may play a role in sphingolipid metabolism reprogramming and may contribute to protecting insects from oxidative stress damage under hypoxic conditions. This may represent a potential approach for enhancing the efficiency of hypoxic-based techniques for stored-product insect control.

## Data Availability

The data presented in this study are available upon request from the corresponding author.

## Supplementary Materials

The following supporting information can be downloaded at: https://www.mdpi.com/article/10.3390/insects17090904/s1, Figure S1. Loading efficiency of *siHIF1α*, *siGFP*; Figure S2. Pearson’s correlation heatmap between the CS-*siHIF1α* group and the CS-*siGFP* group; Table S1. Primers used for qRT-PCR; Table S2. Primers used for full-length *HIF1α* cDNA amplification and sequence verification; Table S3. siRNA sequence GenBank accession numbers of HIF1α protein used in the alignment; Table S4. GenBank accession numbers of HIF1α protein used in the phylogenetic analysis; Table S5. siRNA sequence; Table S6. The full sequence of *CcHIF1α*; Table S7. Relative differences in differential lipid species between CS-siHIF1α groups and CS-siGFP groups.

## Abbreviations

The following abbreviations are used in this manuscript:

CAT	Catalase
POD	Peroxidase
ROS	Reactive Oxygen Species
ACar	Acylcarnitine
Cer-ADS	Ceramide alpha-hydroxy fatty acid-dihydrosphingosine
Cer-BS	Ceramide beta-hydroxy fatty acid-sphingosine
Cer-NDS	Ceramide non-hydroxyfatty acid-dihydrosphingosine
DAG	Diacylglycerol
FAHFA	Fatty acid ester of hydroxyl fatty acid
HBMP	Hemibismonoacylglycerophosphate
LPC	Lysophophatidylcholine
LysoPE	Lysophosphatidylethanolamine
MGDG	Monogalactosyldiacylglycerol
PE	Phosphatidylethanolamine
PG	Phosphatidylglycerol
SHexCer	SulfurHexosylceramide hydroxyfatty acid
SQDG	Sulfoquinovosyl diacylglycerol
*PDK_1_*	pyruvate dehydrogenase kinase
*ACC*	Acetyl-CoA carboxylase
SOD	Superoxide dismutase
GST	Glutathione S-transferase
LPO	Lipid hydroperoxide
Cer	Ceramides
Cer-AS	Ceramide alpha-hydroxy fatty acid-sphingosine
Cer-EOS	Ceramide Esterified omega-hydroxy fatty acid-sphingosine
Cer-NS	Ceramide non-hydroxyfatty acid-sphingosine
DGTS	Diacylglyceryl trimethylhomoserine
GlcADG	Gcuronosyldiacylglycerol
HexCer-AP	Hexosylceramide alpha-hydroxy fatty acid-phytospingosine
LysoPC	Lysophophatidylcholine
MAG	Monoacylglycerol
PC	Phosphatidylcholine
PEtOH	Phosphatidylethanol
PI	Phosphatidylinositol
SM	Sphingomyelin
TAG	Tacylglycerol
*LDH*	lactate dehydrogenase
*FASN*	Fatty acid synthase
