# Supplementary material for: Function of HIF-1α in Regulating Sphingolipid Metabolism and Alleviating Oxidative Stress Damage in Callosobruchus chinensis Under Hypoxia
Source: Insects. 2026 Aug 28;17(9):904. doi: 10.3390/insects17090904 (PMC13607347; doi:10.3390/insects17090904)
Supplement: Supplementary file 1 [file insects-17-00904-s001.zip › insects-4447039-supplementary.pdf]

Supporting information

Function of *HIF1α* on regulating sphingolipid metabolism alleviating oxidative stress damage in *Callosobruchus chinensis* under hypoxia

Xiao Li[2], Sufen Cui*[1], Zhichao Wan[2], Xuemei Sun[1], Yutong Liu[2], Xueqing Geng[3], Yujie Lu[1]

[1]School of Grain Science and Technology, Jiangsu University of Science and Technology, Zhenjiang 212003, China; [2]School of Biotechnology, Jiangsu University of Science and Technology, Zhenjiang 212100, China;[3]School of Agriculture and Biology, Shanghai Jiao Tong University, Shanghai 200240, China.

Table S1. Primers used for qRT-PCR
Table S2. Primers used for full-length *HIF1α* cDNA amplification and sequence verification
Table S3. *siRNA* sequence GenBank accession numbers of HIF1α protein used in the alignment
Table S4. GenBank accession numbers of HIF1α protein used in the phylogenetic analysis
Table S5. *siRNA* sequence
Table S6. The full sequence of *CcHIF1α*
Figure S1. Loading efficiency of *siHIF1α*, *siGFP*.
Figure S2. Pearson's correlation heatmap between the CS-*siHIF1α* group and the CS-*siGFP* group.
Table S7. Relative differences in differential lipid species between CS-*siHIF1α* groups and CS-*siGFP* groups.

* Corresponding author at: School of Grain Science and Technology, Jiangsu University of Science and Technology. Tel: +86-511-85626711; Email: sufen18@just.edu.cn

Table S1 Primers used for RT-qPCR

	5'-3'	
	F	R
CcHIF1α	GAGGACCTGGACAGAATG	GTTGTTCAGCGATGTGTT
CcPDK1	GACCAATGAAGCCAACGAATTACTG	CTGATCTGCCTGTCGCCAATG
CcLDH	GGCGATTCGTCCTTGTCTACAG	TACCGTGCGTCTATGATACATTGATAC
CcSPTLC1	GACCAATGAAGCCAACGAATTACTG	CTGATCTGCCTGTCGCCAATG
CcKDSR	GCCAAGAATGAGATCAAGAGTT	ATTCCAGCACAGTTCACCAG
CcSMPD3	CCACGAAGCTATTGAAGCAA	CCAGAAGAAGGCATACGAAAA
CcSGPL1	AGGCTGAAGTCATAAGAATAGCAG	GAATGAATGGTTGATGGCACTA
CcSGMS2	TGTATCAGCGACAACCACTCTT	AAGCCCAGTCATAAGCAGG
CcDGAT	TTGCCAAATCTGGGACCGT	GCGATACCAAATCACTTAGCCT

Table S2 Primers used for full-length *HIF1α* cDNA amplification and sequence verification

	Numbers	5'-3'	
		F	R
5' Race	Joint primers	GGCCACGCGTCGACTAGTACCCCCCCCCCCCCCC(F1)	GGCCACGCGTCGACTAGTAC(F1)
	Specific primers	CAATCTGTGAAATGCCCAAATAGG(F2)	GTCGCATGACGGAGGCCTTG(R2)
	Nest primers	GGCCACGCGTCGACTAGTACCCCCCCCCCCCCCC	CAATCTGTGAAATGCCCAAATAGG
		GGCCACGCGTCGACTAGT	GTCGCATGACGGAGGCCTTG
3' Race	Joint primers	GACAAGGCCGTGATCCCGAAAGCTTTTTTTTTTTTTTTTTTTTTTTTVN(F1)	GACAAGGCCGTGATCCCGAAAGC(R1)

	Specific primers	TCAACACCGCCATATAGCTGCAG(F2)	GGGGGTGCTGCTGTCCGGTC(R2)
	Nest primers	GACAAGGCCGTGATCCCGAAAGC	TCAACACCGCCATATAGCTGCAG
		GACAAGGCCGTGATCCCGAAAGC	GGGGGTGCTGCTGTCCGGTC
Primers for full-length sequence verification”		TAAGAAGGAGATATACATATGGGTAT GTTTAAAAGCATGGAAAATTCC	TGGTGGTGGTGGTGGTGCTCGAGTATGGCG GTGTTGATGTCGAGAGCTG

Table S3 *siRNA* sequence GenBank accession numbers of HIF1α protein used in the alignment

Gene	specie	Accession number	E-value	Identity (%)
HIF1α	*Homo sapiens*	NP_001521.1	6E-104	49
	Drosophila melanogaster	NP_001263100.1	4e-05	52
	Caenorhabditis elegans	NP_001023891.2	1e-36	30
	Tribolium castaneum	XP_015835862.1	0.0	61

Table S4 GenBank accession numbers of HIF1α protein used in the phylogenetic analysis

Gene	specie	Accession number	E-value	Order
HIF1α	*Callosobruchus maculatus*	AFL70631.1	0.0	Coleoptera
	Anoplophora glabripennis	XP_023312868.1	0.0	Coleoptera
	Aethina tumida	XP_049826586.1	0.0	Coleoptera
	Zophobas morio	XP_063918499.1	0.0	Coleoptera
	Sitophilus oryzae	XP_030765744.1	0.0	Coleoptera
	Phaedon cochleariae	CAH1164562.1	0.0	Coleoptera
	Euwallacea similis	XP_066262737.1	0.0	Coleoptera

Euwallacea fornicatus	XP_066142399.1	0.0	Coleoptera
Tribolium castaneum	XP_015835862.1	0.0	Coleoptera
Tribolium madens	XP_044264881.1	0.0	Coleoptera
Abscondita terminalis	KAF5274064.1	0.0	Coleoptera
Diabrotica virgifera virgifera	XP_028155587.1	0.0	Coleoptera
Cylas formicarius	XP_060536115.1	0.0	Coleoptera
Leptinotarsa decemlineata	XP_023021893.1	0.0	Coleoptera
Diabrotica balteata	CAG9826478.1	0.0	Coleoptera
Dendroctonus ponderosae	XP_019758020.1	0.0	Coleoptera
Psylliodes chrysocephala	CAH1112318.1	0.0	Coleoptera
Hypothenemus hampei	KAL1513012.1	3e-174	Coleoptera
Nicrophorus vespilloides	XP_017778701.1	0.0	Coleoptera

Table S5 *siRNA* sequence

	5'-3'	
	F	R
siHIF1α	CGAUACGCAGAGCAAAAGGCTT	GCCUUUUGCUCUGCGUAUCGTT
siGFP	GGCUACGUCCAGGAGCGCACC	UGCGCUCCUGGACGUAGCCUU

Table S6. The full sequence of *CcHIF1α*

Gene	Full sequence
CcHIF1α	CCCCCCCCCCCGTGTCAGAATGACAGTTACAATATCTGTGAAGAAGTGAACTTGATGGGTATGTTTAAAAG<br>CATGGAAAATTCCATTTCTTATCAAGATCATACGAACTCCCCTCGCGATGATAAGCTTCCAATATACTCTTC<br>TCCAAACATGGAAGAGACTATCCCATATCAGGACAGTGCAAATGTTAGTTTAAGTACGAATCACTTAAATG

	GTTCTTGTGGTAGTGTGAAAACTCGCGTTAAGCCTAATGGGTTATTAGCTAAACTGGGTGTTCCTTGCCAGA
	CTGTTCCGAACTATGACTATGGAAAGTGCTACAGTTACTATGCCAATGGATGTTACAATACTTGCCAGTTCG
	TGGAGATGGGTGACATGGAGGATTTCATGAATAACGAGAAACGCAAGGAGAAATCTCGAGATGCAGCGCG
	ATGCCGCAGGTCGAAAGAGACCGAAATATTCACAGACCTGGCGAAAGCCCTGCCGCTCACAAACGAACAG
	ATCTCACAGCTCGACAAGGCCTCCGTCATGCGACTGGCCATCGCCTATCTCAAAGTCAGAGAAATGGTCGA
	CCTAGTGCCAGCACTAGGAAAATCCGAAAAATGCGAATCCCAGGACGAATCGATACTCCTGAAATCACTCG
	AAGGCTTCATCATCGTACTTTCGAACGATGGCGATATTATATATTTATCAGAAAACGTCAGTACCTATTTGG
	GCATTTCACAGATTGATTTGATGGGTCAGAACGTGTTTGAGTTCAGCCACCCTTGTGATCATGATGAAATCA
	GAGACGTAATTTCCGGGAAATCCCAGGAGGAGTTCAACATTCCCAGATCGATTTTCATCCGTCTGAAGTGT
	ACGCTGACGAATAAAGGAAGATCTGTCAATCTCAAATCTGCTACATATAAGGTTATACATTGTACGGGTCA
	CATGTTACAATCTCCAAGCATAAAGAAAGAGGCTGACGAGACGACAACCAAAGTCCAGAGTTGTTTCTTAG
	CAGTCGGCCAACCAATTCCACATCCGTCAAACATCGAAGCGCCACTTCCAAAGCAAACCTTCCTTACCAAA
	CACAGCTTGGATATGAAGTTCATACACGCCGATGATGAATTCATGAAAAATATACTGGGATACGAACAAAA
	CGACCTGATCGGCAAATCAGTGTACGAATATCACCATGCTATGGACAGCGATGCTATACTGGCTGCCTACA
	AGTGTTTATTTGCAAAAGGACAGTGTGAAACGAACCGCTACCGATTCCTGGCAAAGAGGGGAGGTTACATT
	TGGGTGGTGACACAGGCCACACTAATACATGACAAGATACACAAACCGCATAGCGTTGTCAGCGTCAACTA
	CGTCATAAGCGGTTTAGAATGTAAGAATGAGATTTATTCGGCATTCCAAATTGAATCGGTGATACCGGAGA
	GTAGTGCTAAGGGCGATACAGATAACAACAAGGAGAATCAGGATAGTAGCAATCGGAAGGAGAGTGTTAA
	GGAGGAGGCGAGTGTGGTGCAAGTGAAGGAGAAGGAGAAGGAGCCGGAGCAACAGCCGCCGCTGGAGAA
	ACGGAGGCCGGTGGTGTCGACCTCCAGGTTGTTCGGGGCCGATAGGGGATCGGGCCCTAAGCCCAGAATCG
	CTACCAAGGAGATATTCGGCCCTAGAACGGAGGAGATGAGCAAGGGCTATCTGATGTTCTCCGACGATACG
	CCCGGCTTGACAATGCTGAAAGACGAGCCGGACGATCTGACCCACCTGGCTCCCGTGGCAGGTGATGTTTG
	CGTCCCCTTGGACGACCACCCCTTCCTGGCCGACATGCTGGACGACATCCTGCTCAAGGACAACTTGGCGCC
	CCTCCTGGCGGACGAACCTACCGATCCCTTCATCGCGTACAGGGATCAGGAACCGTCGCCGCAGTTGCTGT
	CGCCCAATATGTCAAAGACGTCCGACAGTCTTCCATCTTTGGGCAGTCCGAGCGACTCGCTGCTCGACGAG

	GACCAGATGTCCACGTTCATGAATCTCCAGATGGACGACGAGACGGGCGACATGACGCTAAGGGCGCCGT<br>ACATCCCCATGGACATCGCCGACGACCTGCCGTTGCTGATGTCCAACGACCTGATGTGGAGCAGCAACGAG<br>AAGAAGCAGCTGCCCGACAACAATTCCAGTTTGGCGCAGCTGCTGTCGTCGTCGATGAACAAGCACGGCCT<br>GAAGGCGAACGACCACGGAGGTGGCGGCGGGGGGTTGGTCGTACCTGAGGAGGACCTGGACAGAATGTTT<br>GGAGATAAAACCGACAAAAGCAAATCGTGGACGACACAGAACAACCTGAAAAACACATCGCTGAACAACC<br>AGCGGATCGAGCGCTCCCCTAACATAAAACGCAGCAGTCCGTCCACGTACGATACGCAGAGCAAAAGGCC<br>GAAGAACGAGCCTAAAGAGAAGATGTCATCCGAACTGCTCCAACAGCTCATGTCCAACAACCACCATCGA<br>GGTAGGCCGAAAGGCAAGAGCAACTGGCTGTTGGACAGCGGCGGCCAAAAGGCGGCCTGCATCTCGCAAC<br>CGAGCGACAGCGTTCTGATGAACCTCCTCGACCCGCCGTTCAGTTCGGCGATGCCGAAACCGCGCGTCACC<br>CCCGGTGACATGCTGATGCGCCAGAAGAAGAAGAGGAAGCTGGAACTGGAACGCGACAAAAGGAACGTG<br>ATGCGTAAGAACTCGCTGTCCCTGTTGGAGCCCGAGGCCAACTGTCTGGCCTCTTTGATGGACCTCACGCAA<br>CACGATTACGAAGTGAACGCCCCCGTCAACGATTACCTGTTGCAGGGCGAGGAACTACTCACAGCTCTCGA<br>CATCAACACCGCCATATAGCTGCAGGGGACGGCACCCTCCGGGGGGTGCTGCTGTCCGGTCACGTTGCGGG<br>AGTACTGCTTGGGTACTAGGAGTATAGTATGATATTATATCGCTAGATCACTATGAAAGACGAAACATACG<br>TTGCACTTCTAGGGCACTAGGAATGTTTCTGAAGCAGGGGGGTGGATTGGCTGGCTCTGACGGTAAAGCTT<br>AGGGCTTACCTTACCTAGCCTACACGGAACATGAGGCTAATGATCATAACAGTCGATGCCCCTGGCGCCAA<br>AATACACAAAAAAAAAAAAAAAAAAA

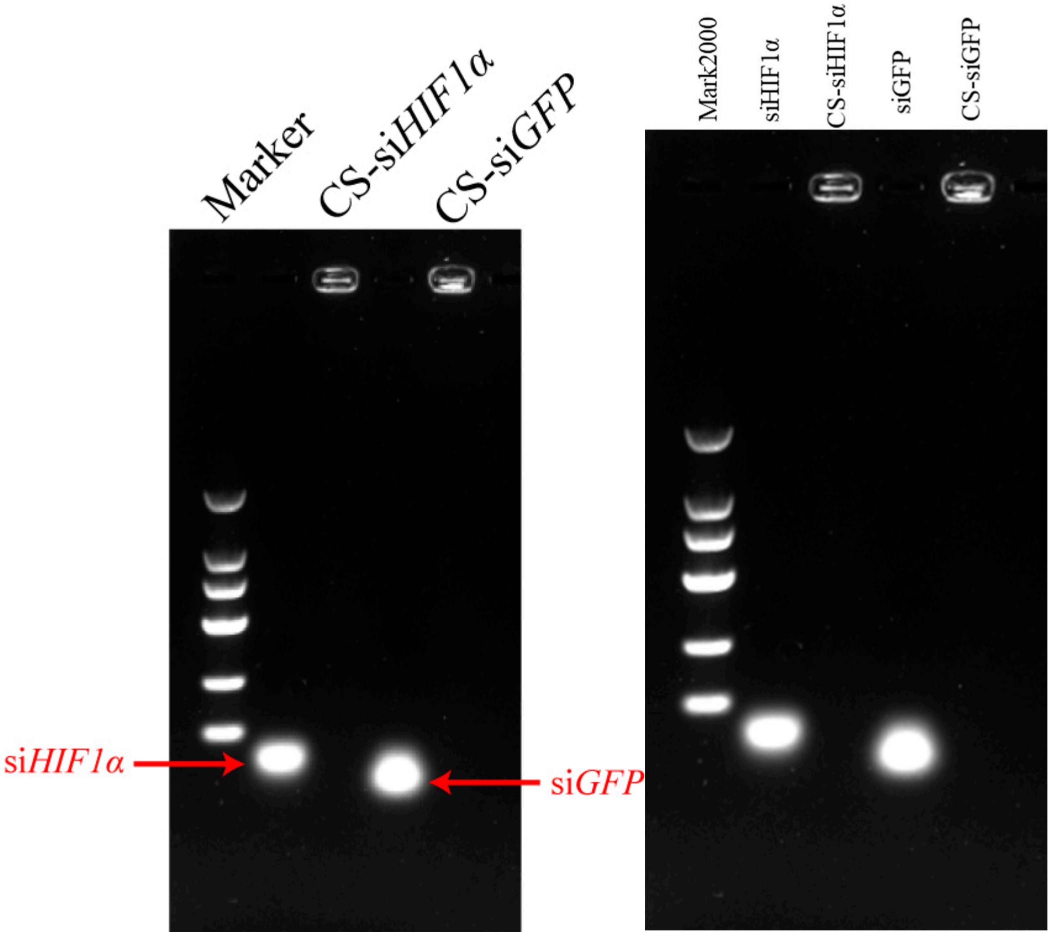

Figure S1. Loading efficiency of *siHIF1α*, *siGFP*. The formation of CS-TPP-dsRNA complexes was verified by agarose gel electrophoresis. Marker2000, naked siRNA and CS-siRNA complexes were resolved on 1% (w/v) agarose gel, stained with loading buffer, and photographed under blue light. The picture of the gel shows differences in the migration of free siRNA and CS-siRNA complexes.

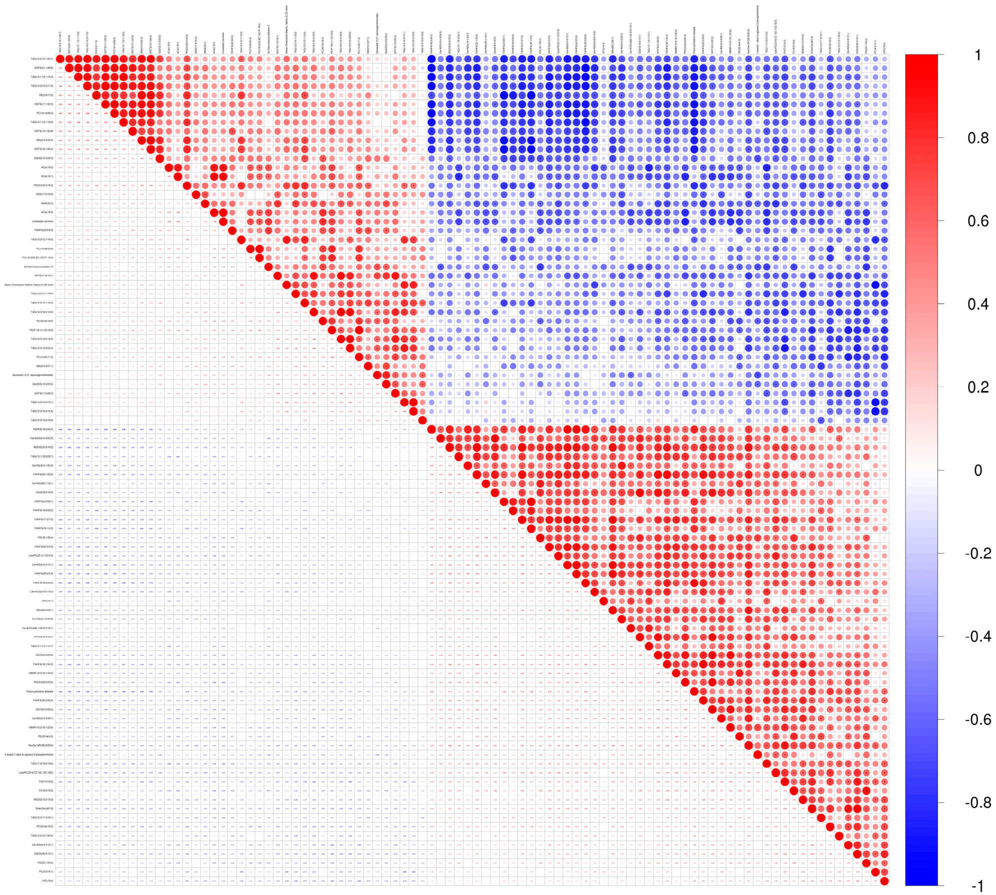

Figure S2. Pearson's correlation heatmap between the CS-*siHIF1α* group and the CS-*siGFP* group. The correlation between the CS-*siHIF1α* group and the CS-*siGFP* group was assessed using the correlation coefficient (r). An r value between 0 and 1 indicates a positive correlation, whereas an r value between -1 and 0 indicates a negative correlation. The absolute value of r reflects the strength of the correlation: the closer |r| is to 1, the stronger the correlation; the closer |r| is to 0, the weaker the correlation. Red and blue gradients indicate positive and negative correlations, respectively ($|r| > 0.5$, $p < 0.05$).

Table S7. Relative differences in differential lipid species between CS-*siHIF1α* groups and CS-*siGFP* groups.

Num	Name	Super.Class	Relative difference(%)	Pathway
1	22-Deoxoisocucurbitacin D	22-Deoxoisocucurbitacin	-44.27949517	
2	2-acetyl-1-alkyl-sn-glycero-3-phosphocholine	2-acetyl-1-alkyl-sn-glycero-3-phosphocholine	77.3140574	Ether lipid metabolism
3	5beta-Cholestane-3alpha,7alpha,24,26-tetrol	5beta-Cholestane-3alpha,7alpha,24,26-tetrol	-32.34555823	
4	ACar(16:1)	ACar	-40.97254996	Thermogenesis
5	ACar(18:2)	ACar	-25.8871674	Thermogenesis
6	ACar(18:3)	ACar	-58.30447881	Thermogenesis
7	Cer(d18:0/18:0)	Cer	26.45674191	Sphingolipid metabolism
8	Cer/ADS(d14:0/20:0)	Cer/ADS	59.33715481	Sphingolipid metabolism
9	Cer/AS(d14:2/12:1)	Cer/AS	138.3058165	Sphingolipid metabolism
10	Cer/AS(d14:2/20:0)	Cer/AS	39.35512935	Sphingolipid metabolism
11	Cer/AS(d14:3/12:1)	Cer/AS	39.10770854	Sphingolipid metabolism
12	Cer/BS(d14:2/38:1)	Cer/BS	67.88337103	Sphingolipid metabolism
13	Cer/EOS(d28:1/23:0/O/18:1)	Cer/EOS	43.76529284	Sphingolipid metabolism
14	Cer/NDS(d19:0/19:0)	Cer/NDS	48.57348801	Sphingolipid metabolism
15	Cer/NS(d14:1/24:2)	Cer/NS	63.14365369	Sphingolipid metabolism
16	Cer/NS(d30:1/18:1)	Cer/NS	51.53810165	Sphingolipid metabolism
17	DAG(17:0/18:2)	DAG	-21.38280245	Glycerophospholipid metabolism
18	DGTS(11:0/26:2)	DGTS	-31.39392129	Glycerophospholipid metabolism

19	DGTS(16:1/26:4)	DGTS	-43.83337768	Glycerophospholipid metabolism
20	DGTS(17:0/18:1)	DGTS	-34.7790692	Glycerophospholipid metabolism
21	DGTS(17:1/20:5)	DGTS	-44.17358069	Glycerophospholipid metabolism
22	DGTS(19:1/22:6)	DGTS	-16.82584462	Glycerophospholipid metabolism
23	DGTS(21:1/22:6)	DGTS	-41.72200227	Glycerophospholipid metabolism
24	DGTS(3:0/22:2)	DGTS	56.60161661	Glycerophospholipid metabolism
25	DGTS(4:0/22:6)	DGTS	39.29610444	Glycerophospholipid metabolism
26	FAHFA(17:2/7:0)	FAHFA	94.58865638	
27	FAHFA(18:1/24:2)	FAHFA	40.44382033	
28	FAHFA(18:1/4:0)	FAHFA	48.04683843	
29	FAHFA(18:2/20:0)	FAHFA	37.3837338	
30	FAHFA(18:3/24:2)	FAHFA	37.74196815	
31	FAHFA(19:0/20:0)	FAHFA	29.33972654	
32	FAHFA(2:0/20:1)	FAHFA	47.72135446	
33	FAHFA(22:1/20:0)	FAHFA	40.32240063	
34	FAHFA(22:2/20:0)	FAHFA	52.38489608	
35	FAHFA(22:2/3:0)	FAHFA	125.7310913	
36	FAHFA(22:5/6:0)	FAHFA	-47.22797916	
37	FAHFA(24:2/3:0)	FAHFA	146.1095336	
38	GlcADG(15:0/23:0)	GlcADG	-51.82673114	
39	HBMP(16:0/18:1/22:0)	HBMP	62.46075274	
40	HBMP(16:2/18:1/22:6)	HBMP	108.0079968	
41	HexCer/AP(t20:0/20:0)	HexCer/AP	171.7324178	
42	Linoelaidyl carnitine	Linoelaidyl	-27.93487538	
43	LPC(14:1)	LPC	94.4807769	

44	LPC(19:0)	LPC	307.278714	
45	LysoPC(22:4(7Z,10Z,13Z,16Z))	LysoPC	66.16006262	
46	LysoPE(20:1(11Z)/0:0)	LysoPE	36.68312277	
47	MAG(22:3)	MAG	-67.55358529	Glycerolipid metabolism
48	HBMP(13:0/13:0)	MGDG	57.92023606	
49	MGDG(20:0/18:2)	MGDG	87.53837962	
50	PC(14:0e/17:2)	PC	-22.38750811	Glycerophospholipid metabolism
51	PC(14:0e/22:4)	PC	-20.41522033	Glycerophospholipid metabolism
52	PC(16:1e/26:2)	PC	-33.77012476	Glycerophospholipid metabolism
53	PC(18:3(6Z,9Z,12Z)/P-18:0)	PC	-21.00616657	Glycerophospholipid metabolism
54	PC(20:3e/18:2)	PC	-27.85724595	Glycerophospholipid metabolism
55	PC(20:5e/18:2)	PC	87.93761716	Glycerophospholipid metabolism
56	PC(4:0/14:1)	PC	77.3025244	Glycerophospholipid metabolism
57	PE(22:6e/4:0)	PE	31.80512152	Glycerophospholipid metabolism
58	PE(3:0/17:0)	PE	-63.57884372	Glycerophospholipid metabolism
59	PE(P-18:1(11Z)/16:0)	PE	-24.36421222	Glycerophospholipid metabolism
60	PEtOH(23:0/18:2)	PEtOH	-36.16290168	
61	PEtOH(26:0/20:5)	PEtOH	55.96159163	
62	PG(18:1/26:4)	PG	38.006736	Glycerophospholipid metabolism
63	PG(22:1/20:4)	PG	80.66072468	Glycerophospholipid metabolism
64	PI(16:0/19:0)	PI	50.8079382	Glycerophospholipid metabolism
65	PI(27:0/18:2)	PI	265.2885736	Glycerophospholipid metabolism
66	Polyoxyethylene dioleate	Polyoxyethylene	43.67129845	
67	SHexCer(d47:0)	SHexCer	104.2556557	

68	SM(d14:0/30:2)	SM	-45.17345307	Sphingolipid metabolism
69	SM(d14:2/27:1)	SM	-36.00542376	Sphingolipid metabolism
70	SM(d28:3/38:1)	SM	2652.95897	Sphingolipid metabolism
71	Spinacetin 3-(2''-apiosylgentiobioside)	Spinacetin	-39.28818023	
72	SQDG(12:0/23:0)	SQDG	-36.18104444	Glycerolipid metabolism
73	SQDG(18:0/19:1)	SQDG	114.447723	Glycerolipid metabolism
74	SQDG(26:0/19:1)	SQDG	258.8778496	Glycerolipid metabolism
75	TAG(12:0/12:0/22:2)	TAG	-27.46177941	Glycerolipid metabolism
76	TAG(12:0/12:1/16:0)	TAG	-30.33205007	Glycerolipid metabolism
77	TAG(12:0/13:0/18:2)	TAG	-19.13536633	Glycerolipid metabolism
78	TAG(12:0/13:1/16:0)	TAG	-29.55321385	Glycerolipid metabolism
79	TAG(12:0/13:1/22:7)	TAG	-49.17656488	Glycerolipid metabolism
80	TAG(12:0/14:1/18:0)	TAG	-26.8245147	Glycerolipid metabolism
81	TAG(12:0/16:0/16:5)	TAG	-24.28887007	Glycerolipid metabolism
82	TAG(12:1/12:1/12:1)	TAG	67.82968381	Glycerolipid metabolism
83	TAG(12:1/12:1/19:0)	TAG	-53.08828295	Glycerolipid metabolism
84	TAG(12:2/13:1/22:4)	TAG	31.54172524	Glycerolipid metabolism
85	TAG(13:1/13:1/18:5)	TAG	-41.07047758	Glycerolipid metabolism
86	TAG(13:1/18:3/22:7)	TAG	48.20384622	Glycerolipid metabolism
87	TAG(14:0/14:0/15:1)	TAG	-21.46558805	Glycerolipid metabolism
88	TAG(14:2/14:2/17:3)	TAG	-44.10057918	Glycerolipid metabolism
89	TAG(15:2/17:3/19:1)	TAG	163.78773	Glycerolipid metabolism
90	TAG(17:2/19:0/19:5)	TAG	64.61501172	Glycerolipid metabolism
91	TAG(18:2/18:2/18:3)	TAG	-38.82316552	Glycerolipid metabolism
92	TAG(18:2/18:3/18:3)	TAG	-31.92853584	Glycerolipid metabolism

Note: Relative difference (%): A zero value denotes equal content in both groups; a positive value signifies higher content in the CS-*siHIF1α* group, and a negative value indicates higher content in the CS-*siGFP* group.
